# Detecting outliers when fitting data with nonlinear regression – a new method based on robust nonlinear regression and the false discovery rate

**DOI:** 10.1186/1471-2105-7-123

**Published:** 2006-03-09

**Authors:** Harvey J Motulsky, Ronald E Brown

**Affiliations:** 1 GraphPad Software, Inc., San Diego, CA, USA; 2 AISN Software Inc., Florence, OR, USA

## Abstract

**Background:**

Nonlinear regression, like linear regression, assumes that the scatter of data around the ideal curve follows a Gaussian or normal distribution. This assumption leads to the familiar goal of regression: to minimize the sum of the squares of the vertical or Y-value distances between the points and the curve. Outliers can dominate the sum-of-the-squares calculation, and lead to misleading results. However, we know of no practical method for routinely identifying outliers when fitting curves with nonlinear regression.

**Results:**

We describe a new method for identifying outliers when fitting data with nonlinear regression. We first fit the data using a robust form of nonlinear regression, based on the assumption that scatter follows a Lorentzian distribution. We devised a new adaptive method that gradually becomes more robust as the method proceeds. To define outliers, we adapted the false discovery rate approach to handling multiple comparisons. We then remove the outliers, and analyze the data using ordinary least-squares regression. Because the method combines robust regression and outlier removal, we call it the ROUT method.

When analyzing simulated data, where all scatter is Gaussian, our method detects (falsely) one or more outlier in only about 1–3% of experiments. When analyzing data contaminated with one or several outliers, the ROUT method performs well at outlier identification, with an average False Discovery Rate less than 1%.

**Conclusion:**

Our method, which combines a new method of robust nonlinear regression with a new method of outlier identification, identifies outliers from nonlinear curve fits with reasonable power and few false positives.

## Background

Nonlinear regression, like linear regression, assumes that the scatter of data around the ideal curve follows a Gaussian or normal distribution. This assumption leads to the familiar goal of regression: to minimize the sum of the squares of the vertical or Y-value distances between the points and the curve. However, experimental mistakes can lead to erroneous values – outliers. Even a single outlier can dominate the sum-of-the-squares calculation, and lead to misleading results.

Identifying outliers is tricky. Even when all scatter comes from a Gaussian distribution, sometimes a point will be far from the rest. In this case, removing that point will reduce the accuracy of the results. But some outliers are the result of an experimental mistake, and so do not come from the same distribution as the other points. These points will dominate the calculations, and can lead to inaccurate results. Removing such outliers will improve the accuracy of the analyses.

Outlier elimination is often done in an *ad hoc *manner. With such an informal approach, it is impossible to be objective or consistent, or to document the process.

Several formal statistical tests have been devised to determine if a value is an outlier, reviewed in [1]. If you have plenty of replicate points at each value of X, you could use such a test on each set of replicates to determine whether a value is a significant outlier from the rest. Unfortunately, no outlier test based on replicates will be useful in the typical situation where each point is measured only once or several times. One option is to perform an outlier test on the entire set of residuals (distances of each point from the curve) of least-squares regression. The problem with this approach is that the outlier can influence the curve fit so much that it is not much further from the fitted curve than the other points, so its residual will not be flagged as an outlier.

Rather than remove outliers, an alternative approach is to fit all the data (including any outliers) using a robust method that accommodates outliers so they have minimal impact [2,3]. Robust fitting can find reasonable best-fit values of the model's parameters but cannot be used to compare the fits of alternative models. Moreover, as far as we know, no robust nonlinear regression method provides reliable confidence intervals for the parameters or confidence bands for the curve. So robust fitting, alone, is a not yet an approach that can be recommended for routine use.

As suggested by Hampel [2] we combined robust regression with outlier detection. It follows three steps.

1. Fit a curve using a new robust nonlinear regression method.

2. Analyze the residuals of the robust fit, and determine whether one or more values are outliers. To do this, we developed a new outlier test adapted from the False Discovery Rate approach of testing for multiple comparisons.

3. Remove the outliers, and perform ordinary least-squares regression on the remaining data.

We describe the method in detail in this paper and demonstrate its properties by analyzing simulated data sets. Because the method combines Robust regression and outlier removal, we call it the ROUT method.

## Results

### Brief description of the method

The Methods section at the end of the paper explains the mathematical details. Here we present a nonmathematical overview of how both parts of the ROUT method (robust regression followed by outlier identification) work.

#### Robust nonlinear regression

The robust fit will be used as a 'baseline' from which to detect outliers. It is important, therefore, that the robust method used give very little weight to extremely wild outliers. Since we anticipate that this method will often be used in an automated way, it is also essential that the method not be easily trapped by a false minimum and not be overly sensitive to the choice of initial parameter values. Surprisingly, we were not able to find an existing method of robust regression that satisfied all these criteria.

**Figure 1:**
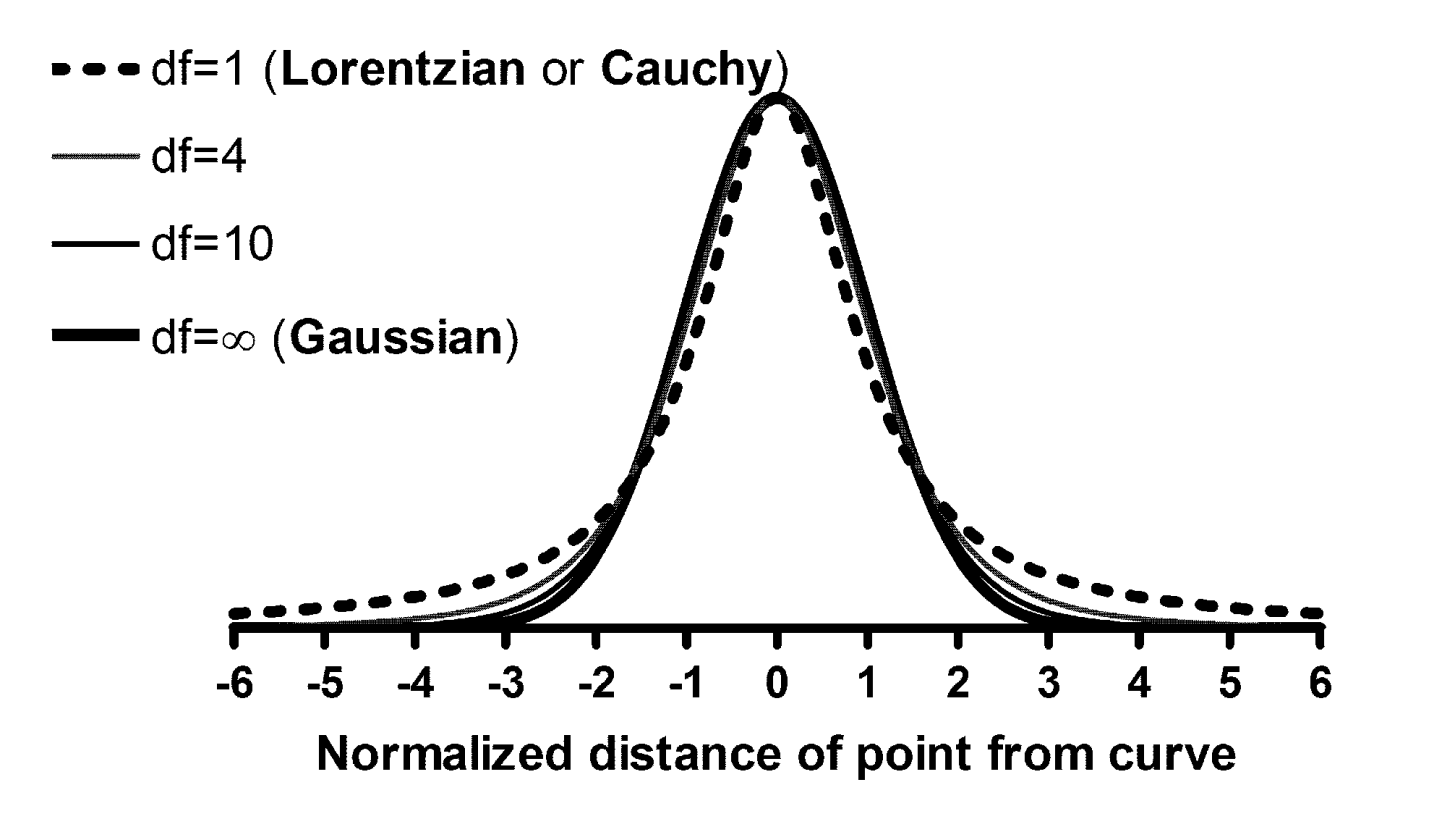
**The Lorentzian distribution**. The graph shows the t probability distribution for 1, 4, 10 and infinite degrees of freedom. The distribution with 1 df is also known as the Lorentzian or Cauchy distribution. Our robust curve fitting method assumes that scatter follows this distribution.

Based on a suggestion in *Numerical Recipes *[4], we based our robust fitting method on the assumption that variation around the curve follows a Lorentzian distribution, rather than a Gaussian distribution. Both distributions are part of a family of t distributions as shown in Figure 1. The widest distribution in that figure, the t distribution for df = 1, is also known as the *Lorentzian *distribution or *Cauchy *distribution. The Lorentzian distribution has wide tails, so outliers are fairly common and therefore have little impact on the fit.

**Figure 2:**
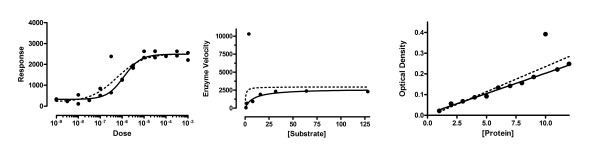
**Robust curve fits vs. least-squares curve fits**. The three examples show that a single outlier greatly affects the least-squares fit (dotted), but not the robust fit (solid).

We adapted the Marquardt nonlinear regression algorithm to accommodate the assumption of a Lorentzian (rather than Gaussian) distribution of residuals. Figure 2 shows three data sets which include an outlier. The solid curves show the results of our robust nonlinear regression, which are barely influenced by the outlier. In contrast, the dotted curves show the least-squares results, which are dramatically influenced by the outlier.

Our method fits data nearly as quickly as ordinary nonlinear regression, with no additional choices required. The robust fitting method reports the best-fit values of the parameters, but does not report standard errors or confidence intervals for these values.

Least-squares regression quantifies the scatter of data around the curve by reporting S_y.x_, sometimes called S_e_, the standard error of the fit. It is computed by taking the square root of the ratio of the sum-of-squares divided by the number of degrees of freedom (N-K, where N is the number of data points and K is the number of adjustable parameters). S_y.x _is in the same units as the Y values, and can be thought of as the standard deviation of the residuals.

The presence of an outlier would greatly increase the value of S_y.x_. Therefore, we use an alternative method to quantify the scatter of points around the curve generated by robust nonlinear regression. We quantify the scatter by calculating the 68.27 percentile of the absolute values of the residuals (because 68.27% of values in a Gaussian distribution lie within one standard deviation of the mean). We call this value (with a small-N correction described in the Methods section) the Robust Standard Deviation of the Residuals (RSDR).

### Outlier detection

After fitting a curve using robust nonlinear regression, a threshold is needed for deciding when a point is far enough from the curve to be declared an outlier. We reasoned that this is very similar to the problem of looking at a set of many P values and choosing a threshold for deciding when a P value is small enough to be declared 'statistically significant'.

When making multiple statistical comparisons, where do you draw the line between 'significant' and 'not significant'? If you use the conventional 5% significance level for each comparison, without adjusting for multiple comparisons, you'll get lots of false positives. The Bonferroni method uses a lower cut-off defined as 5% divided by the number of comparisons in the family. It is a helpful tool when you are making a few comparisons, but is less useful when you make many comparisons as it can miss many real findings (in other words, it has little statistical power). Benjamani and Hochberg [5,6] developed a method to deal with multiple comparisons that takes into account not only the number of comparisons but the distribution of P values within the set. When using this method, you set the value Q in order to control the False Discovery Rate (FDR). If Q is set to 1%, you can expect fewer than 1% of the 'statistically significant' findings (discoveries) to be false positives, while the rest (more than 99%) are real.

We adapted the concept of FDR to create a novel approach to identify one or several outliers. First divide each residual by the RSDR. This ratio approximates a t distribution, which can be used to obtain a two-tailed P value. Now use the FDR method to determine which of these P values is 'significant', and define the corresponding points to be outliers.

### Choosing an appropriate value for Q

Outlier elimination by the FDR technique requires that you choose a value of Q. If Q is small, then very few good points will be mistakenly identified as 'outliers', but the power to detect true outliers will be low. If Q is larger, then the power to find true outliers is high, but more good points will be falsely identified as outliers.

**Figure 3:**
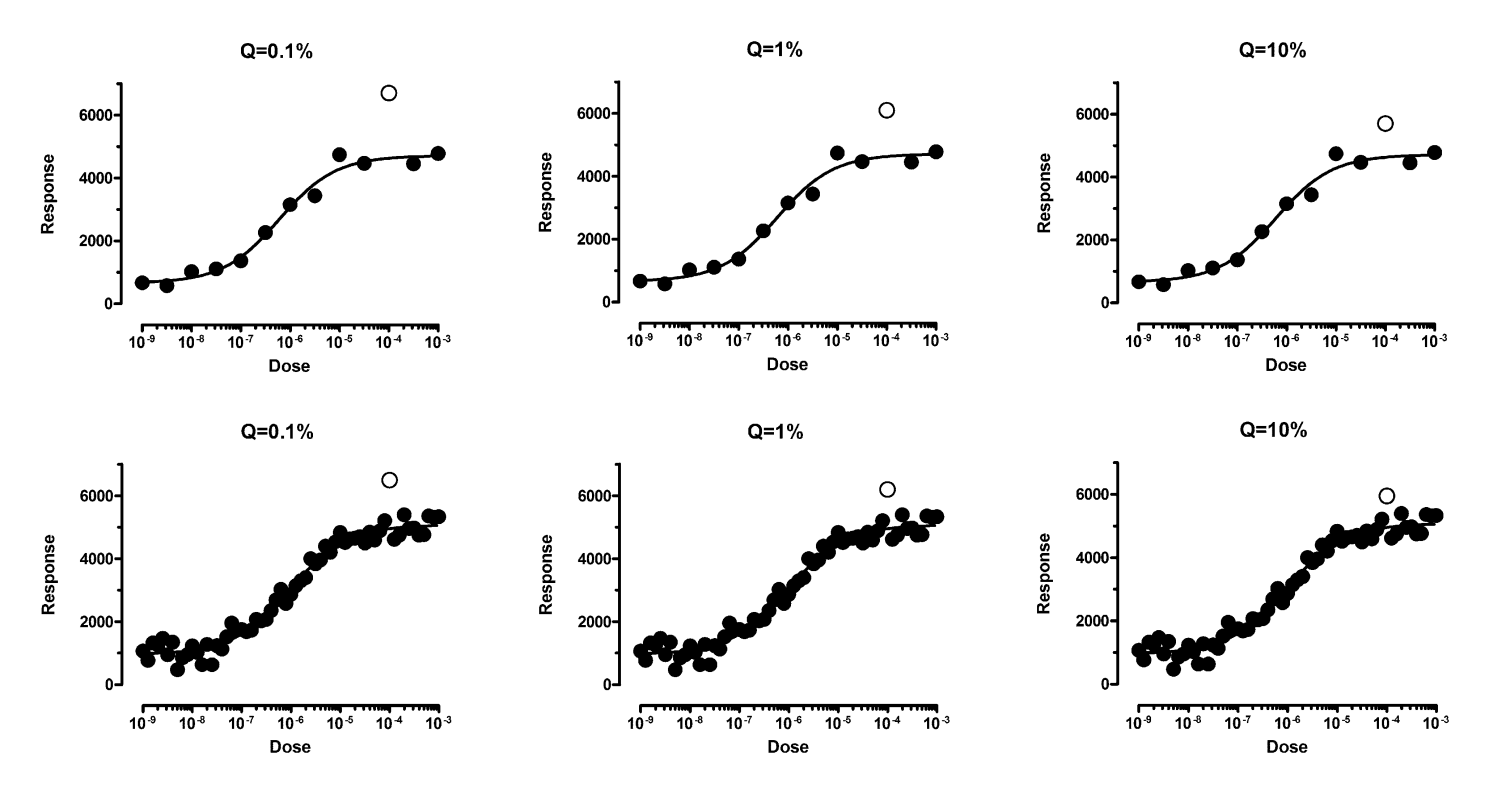
**Choosing a value for Q**. The value of Q determines how aggressively the method will remove outliers. This figure shows three possible values of Q with small and large numbers of data points. Each graph includes an open symbols positioned just far enough from the curve to be barely defined as an outlier. If the open symbols were moved any closer to the curve, they would no longer be defined to be outliers. If Q is set to a low value, fewer good points will be defined as outliers, but it is harder to identify outliers. The left panel shows Q = 0.1%, which seems too low. If Q is set to a high value, it is easier to identify outliers but more good points will be identified as outliers. The right panel shows Q = 10%. We recommend setting Q to 1% as shown in the middle panels.

The graphs in Figure 3 show the consequence of setting Q to 0.1%, 1% or 10%. In each of the graphs, there is one outlier (open symbol) that is placed just beyond the boundary of outlier detection. In every case, if the point were moved a tiny bit closer to the curve, it would no longer be detected as an outlier.

If Q is set to 10%, outlier removal seems a bit too aggressive. The open circles in the right panels are not all that far from the other points. If Q is set to 0.1%, as shown on the two graphs in the left, outlier removal seems a bit too timid. Points have to be pretty far from the rest to be detected. The two graphs in the middle panels have Q set to 1%. The choice is subjective, but we choose to set Q to 1%, although there may be situations where it makes sense to set Q to a lower or higher value.

### If all scatter is Gaussian, how many 'outliers' are mistakenly identified?

We simulated 18 different situations (different models, different numbers of parameters, different numbers of data points). For each, we simulated 10,000 data sets adding Gaussian error, analyzed the results with the ROUT method (setting Q to 1%), and recorded how many outliers were (mistakenly) eliminated. The fraction of data points eliminated as "outliers" ranged from 0.95% to 3.10%, with a median of 1.5%. We observed no obvious correlation between fraction of points eliminated as 'outliers', and the choice of model, sample size, or amount of scatter but we did not investigate these associations in depth.

### How well are single outliers detected?

**Figure 4:**
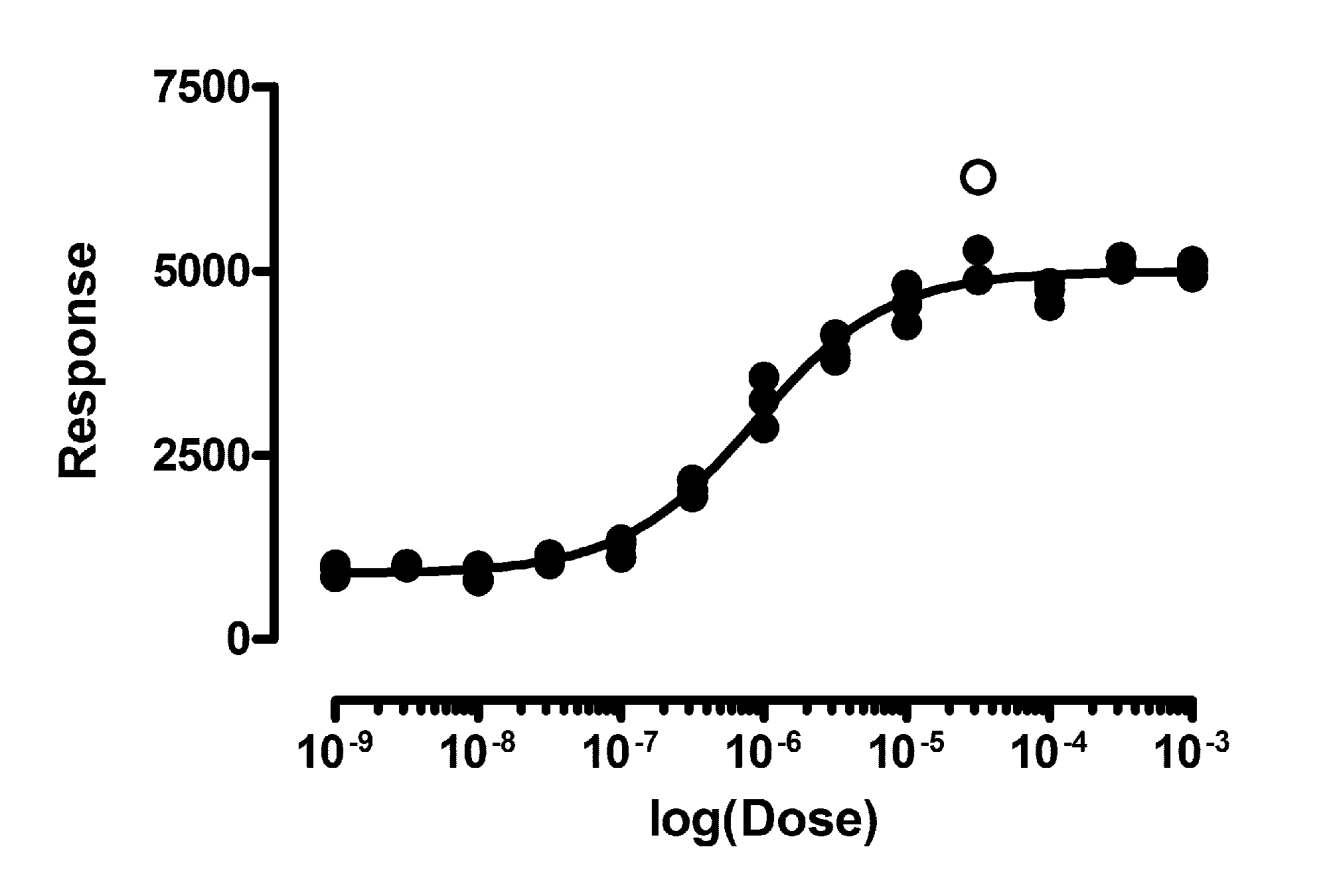
**Identifying extreme outliers**. This shows the first of 5000 simulated data sets with a single outlier (open symbol) whose distance from the ideal curve equations 7 times the standard deviation of the Gaussian scatter of the rest of the points. Our method detected an outlier like this in all but 5 of 5000 simulated data sets, while falsely defining very few good points to be an outlier (False Discovery Rate = 1.18%).

Figure 4 shows a situation where one of the data points is much further from the curve than the rest. We simulated the scatter in 5000 experiments like this by generating Gaussian scatter with a standard deviation of 200, and adding a single outlier that was 1400 Y units away from the curve (shown as an open circle).

The ROUT method (with Q set to 1%) detected the outlier in all but 5 of these simulations. In addition, it occasionally falsely identified some 'good' points as outliers. It identified one point to be an outlier in 95 simulated experiments, two points in 14 experiments, three points in one experiment, four points in one experiment, and five points in one experiment. For each of the simulated experiments, we expressed the false discovery rate (FDR) as the number of 'good points' falsely classified as outliers divided by the total number of outliers detected. For the few experiments where no outliers were detected, the FDR is defined to equal 0.0. The average FDR for the 5000 simulated experiments was 1.18%.

**Figure 5:**
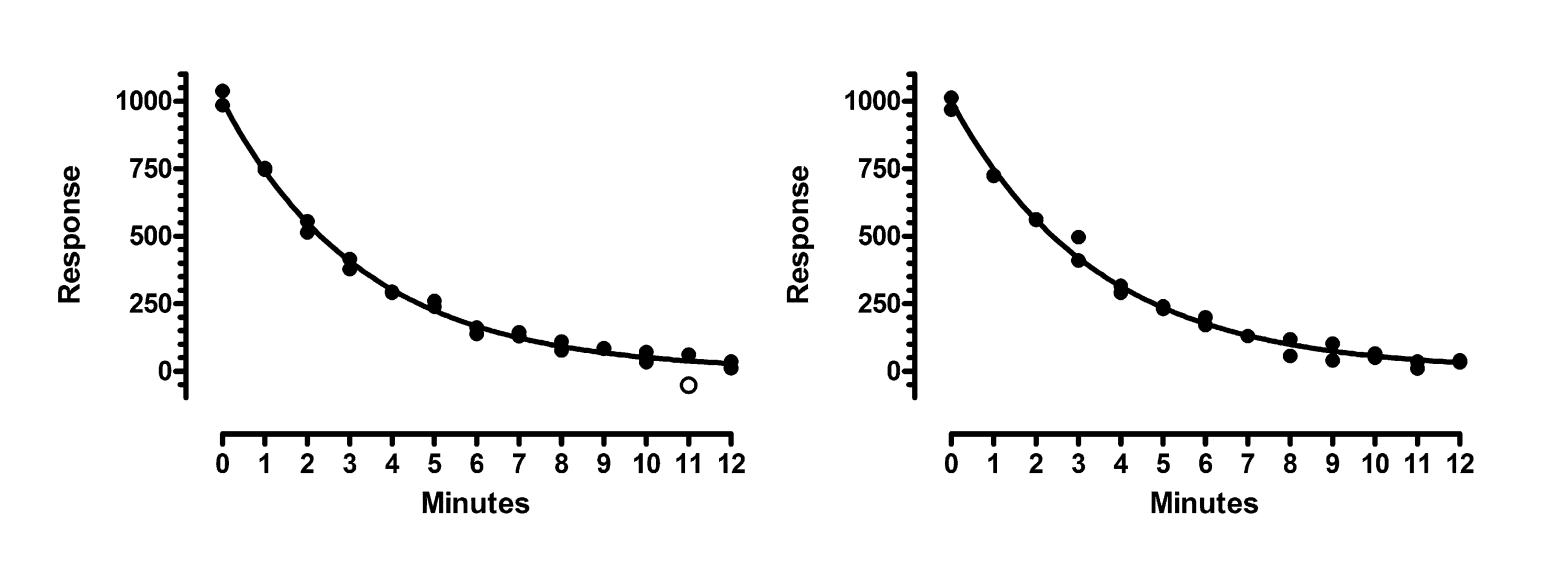
**Identifying moderate outliers**. These are the first two of 5000 simulated data sets, where the scatter is Gaussian but one outlier was added whose distance from the ideal curve equalled 4.5 times the standard deviation used to simulate the remaining points. Our method detected the outlier in the left panel (with Q set to 1%), and in 58% of 5000 simulations, but did not detect it in the right panel or in 42% of simulations. The False Discover Rate was 0.94%.

Figure 5 shows the first two of a different series of simulations. Here the distance of the outlier from the curve equals 4.5 times the standard deviation of the Gaussian scatter. This makes the outlier harder to detect. The left panel shows a simulation where the outlier was detected (open circle). In the right panel, the outlier at X = 3 minutes was not detected.

In 5000 simulations, the ROUT method (with Q set to 1%) detected the outlier in 58.3% of the simulations. Additionally, it mistakenly identified a 'good' point as an outlier in 78 simulated experiments, and two points in 10, and three points in 2. The average FDR was 0.94%. This example shows that our method can also detect moderate outliers most of the time, maintaining the false discovery rate below the value of Q we set.

### How well are multiple outliers detected?

We used the same setup as Figure 4 (the distance of the outliers from the curve equals 7 times the SD of the Gaussian scatter). When we simulated 5000 data sets with nine (of 36 points) being outliers, the ROUT method (with Q set to 1%) detected 86% of the outliers, with an average FDR of 0.06%. With two outliers, it detected more than 99 % of them, with an average FDR of 0.83%.

We also simulated experiments with moderate outliers, using the same setup as Figure 5. When we simulated 5000 experiments with two outliers, 57% of the outliers were detected with an FDR of 0.47%. When we simulated experiments where 5 of the 26 points were outliers, 28% of the outliers were detected with an FDR of only 0.02%.

### How well does it work when scatter is not Gaussian?

**Figure 6:**
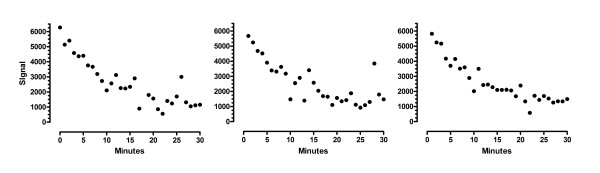
**Simulated data sets where the scatter follows a t distribution with 2 degrees of freedom**. These are the first three of 1000 simulated data sets, where the scatter was generated using a t distribution with 2 degrees of freedom. Note that the data are much more spread out than they would have been had they been simulated from a Gaussian distribution.

Even though the ROUT method was developed to analyze data where the scatter is Gaussian with the addition of a few outliers, we wanted to know if it also works well when the scatter is not Gaussian. We simulated 1000 data sets where the scatter follows a *t *distribution with two degrees of freedom. Figure 6 shows three of these data sets, showing the wide scatter.

**Figure 7:**
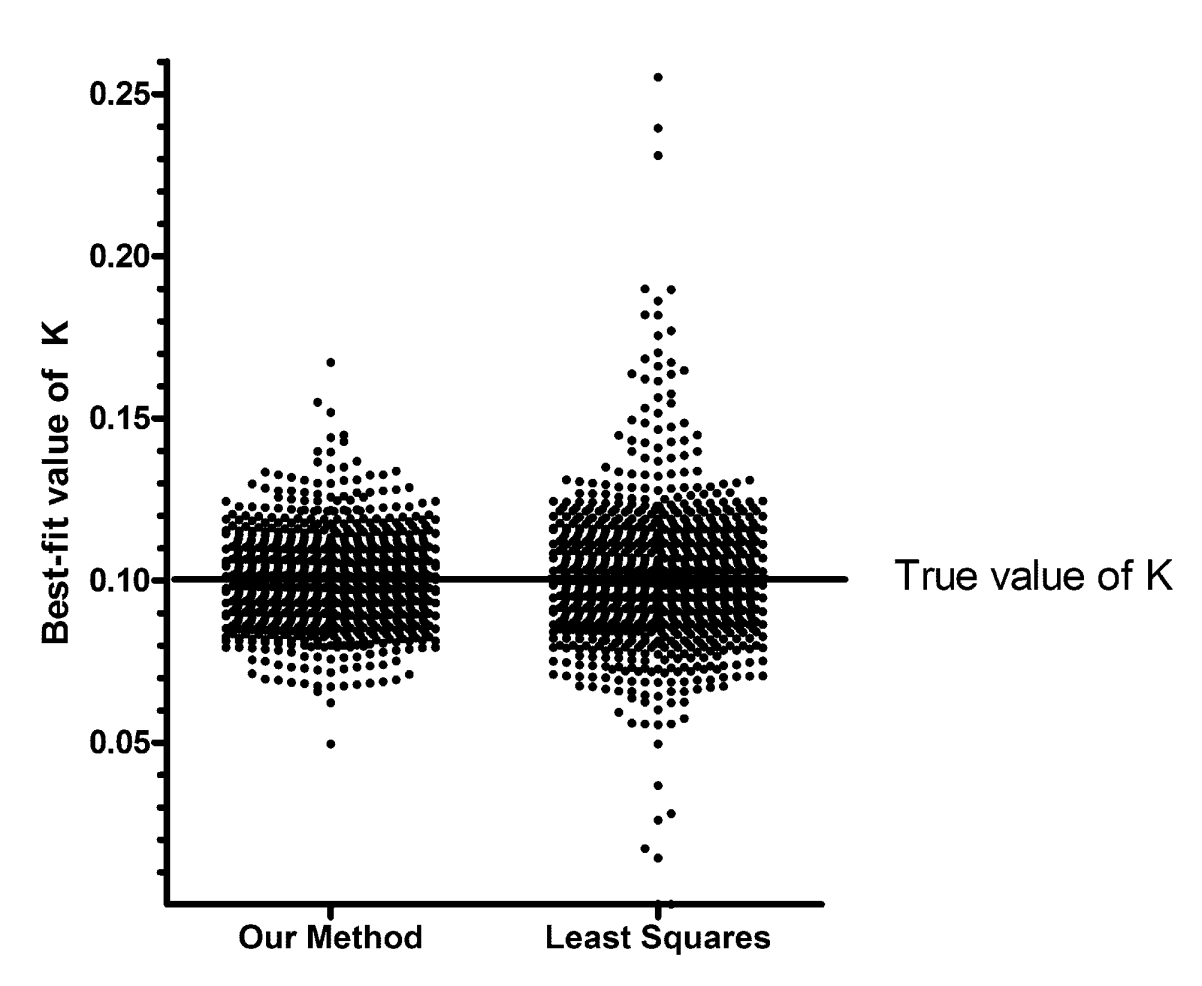
**Best-fit value for the rate constants**. One thousand simulated data sets (similar to those of Figure 6, with scatter much wider than Gaussian) were fit to a one-phase exponential decay model with our method (left) or least-squares regression (right). Each dot is the best-fit value of the rate constant for one simulated data set. The dots are more tightly clustered around the true value of 0.10 in the left panel, showing that our outlier-removal method gives more accurate results (on average) than least-squares regression.

Figure 7 shows the best-fit value of the rate constant k, for 1000 simulated data sets analyzed either by ordinary least-squares regression or by our outlier-removal method. Both methods find the correct rate constant (K = 0.1) on average. But the scatter among individual simulations is much greater with standard least squares regression than with the ROUT method, which has a smaller average error.

### Is the method fooled by garbage data?

**Figure 8:**
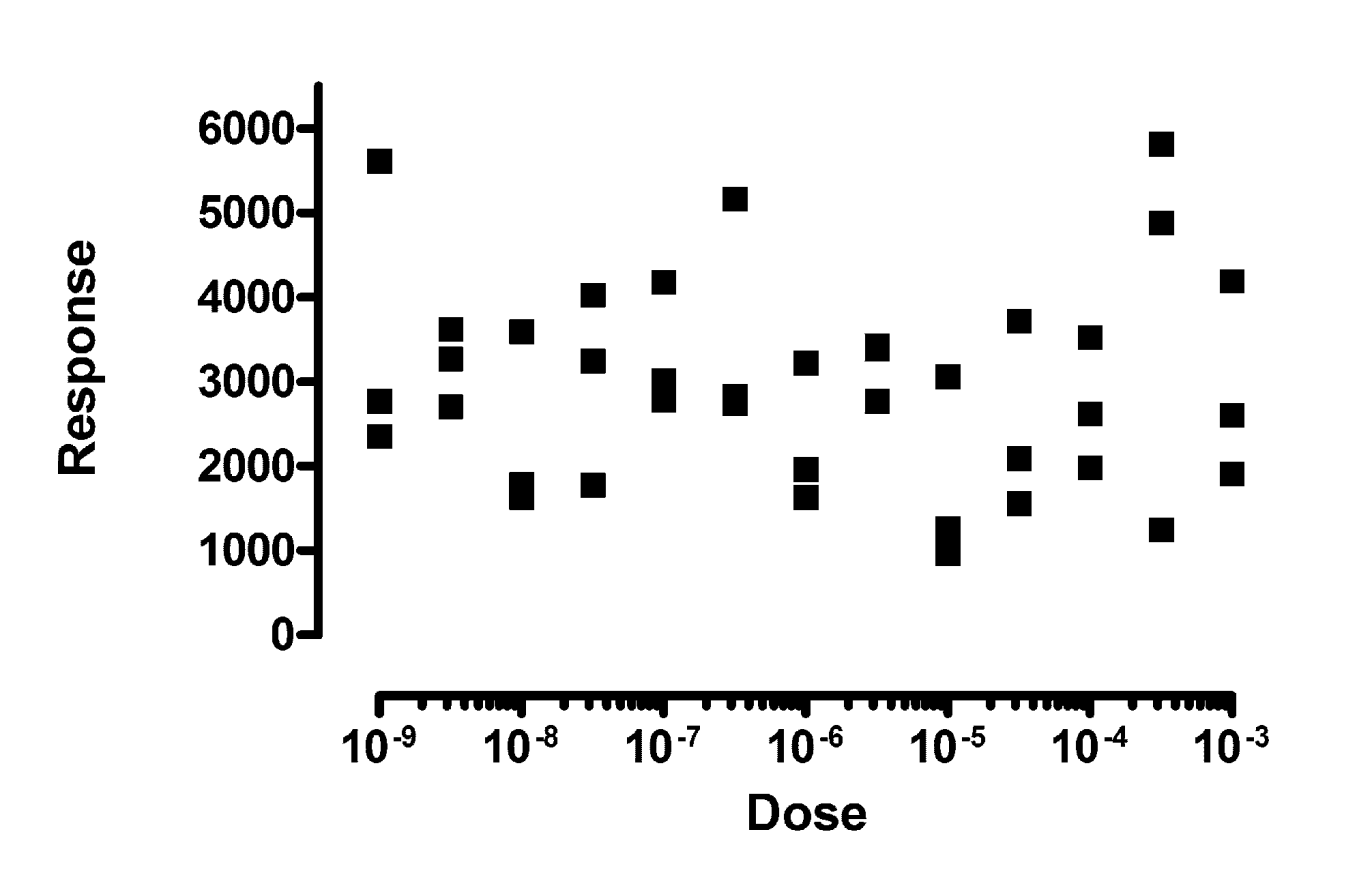
**The ROUT method is not fooled by totally random data**. These data were simulated from a Gaussian distribution around a horizontal line. Each simulated data set was then fit to a sigmoid dose-response curve, fixing the bottom plateau and slope, and fitting the top plateau and the EC_50_. Our fear was that our method would define many points to 'outliers' and leave behind points that define a dose-response curve. That didn't happen. Our method found an outlier in only one of 1000 simulations.

One fear is that an automated outlier rejection method might report seemingly valid results from data that is entirely random with no trend at all. Figure 8 shows the first of 1000 simulated data sets where we tested this idea.

We fit these simulated data sets to a sigmoid dose-response curve, fixing the bottom plateau and slope, asking the program to fit the top plateau and EC50. Both curve fitting methods (least squares, or robust followed by outlier elimination with Q set to 1%) were able to fit curves to about two thirds of the simulated data sets, but the majority of these had EC50 values that were outside the range of the data. Only 132 of the simulated data sets had EC50 values within the range of the data (fit either with robust or ordinary regression), and all the R^2 ^values were less than 0.32. Our outlier removal method found an outlier in only one of these 132 simulated data sets.

These simulations show that the ROUT method does not go wild rejecting outliers, sculpting completely random data to fit the model. When given garbage data, the outlier rejection method very rarely finds outliers, and so almost always reports the same results (or lack of results) as least-squares regression.

### What about tiny data sets?

Another fear is that the outlier removal method would be too aggressive with tiny data sets. To test this, we simulated small data sets fitting one parameter (the mean) or four parameters (variable slope dose-response curve). In both cases, when the number of degrees of freedom was 1 or 2 (so N was 2 or 3 for the first case, and 5 or 6 for the second), our method never found an outlier no matter how far it was from the other points. When there were three degrees of freedom, the method occasionally was able to detect an outlier, but only when it was very far from the other points and when those other points had little scatter.

These simulations show that the ROUT method does not wildly reject outliers from tiny data sets.

## Discussion

### Overview

Ordinary least-squares regression is based on the assumption that the scatter of points around a fitted line or curve follows a Gaussian distribution. Outliers that don't come from that distribution can dominate the calculations and lead to misleading results. If you choose to leave the outliers in the analysis, you are violating one of the assumptions of the analysis, and will obtain inaccurate results.

A widely accepted way to deal with outliers is to use robust regression methods where outliers have little influence. The most common form of robust regression is to iteratively weight points based on their distance from the curve. This method is known as IRLS (iteratively reweighted least-squares). This method is popular because it can be easily implemented on top of existing weighted non-linear least-squares fitting algorithms. The drawback of most IRLS methods is that the weighting schemes correspond to underlying distribution densities that are highly unlikely to occur in practice. For this reason, we chose not to use IRLS fitting, but instead to use a maximum likelihood fitting procedure assuming that scatter follows a Lorentzian distribution density. The best-fit parameters from this approach are sometimes called m-estimates.

Robust methods are appealing because outliers automatically fade away, and so there is no need to create a sharp borderline between 'good' points and outliers. But using robust methods creates two problems. One is that while robust methods report the best-fit value of the parameters, they do not report reliable standard errors or confidence intervals of the parameters. We sought a method that offered reliable confidence intervals without resorting to computationally expensive bootstrapping, but were unable to find an accurate method, even when given data with only Gaussian scatter.

The other problem is that robust methods cannot be readily extended to compare models. In many fields of science, the entire goal of curve fitting is to fit two models and compare them. This is done by comparing the goodness-of-fit scores, accounting for differences in number of parameters that are fit. This approach only works when both models 'see' the same set of data. With robust methods, points are effectively given less weight when they are far from the curve. When comparing models, the distance from the curve is not the same for each model. This means that robust methods might give a particular point a very high weight for one model and almost no weight for a different model, making the model comparison invalid.

We decided to use the approach of identifying and removing outliers, and then performing least-squares nonlinear regression. We define outliers as points that are 'too far' from the curve generated by robust nonlinear regression. We use the curve fit by robust nonlinear regression, because that curve (unlike a least-squares curve) is not adversely affected by the outliers.

Since outliers are not generated via any predictable model, any rule for removing outliers has to be somewhat arbitrary, If the threshold is too strict, some rogue points will remain. If the threshold is not strict enough, too many good points will be eliminated. Many methods have been developed for detecting outliers, as reviewed in [1]. But most of these methods can only be used to detect a single outlier, or can detect multiple outliers only when you state in advance how many outliers will exist.

We adapted the FDR approach to multiple comparisons, and use it as a method to detect any number of outliers. We are unaware of any other application of the FDR approach to outlier detection.

The FDR method requires that you set a value for Q. We choose to set Q to 1%. Ideally, this means that if all scatter is Gaussian, you would (falsely) declare one or more data points to be outliers in 1% of experiments you run. In fact, we find that about 1–3% of simulated experiments had one (or rarely more than one) false outlier.

Why do we find that outliers are identified in 1–3% of simulated experiments with only Gaussian scatter, when Q is set to 1%? The theory behind the FDR method predicts that one or more 'outliers' will be (falsely) identified in Q% of experiments. But this assumes that the ratio of the residual to the RSDR follows a t distribution, so the P values are randomly spaced between 0 and 1. You'd expect this if you look at the residuals from least-squares regression and divide each residual by the Sy.x. Our simulations (where all scatter is Gaussian) found that the average RSDR from robust regression matches the average Sy.x from least-squares regression. But the spread of RSDR values (over many simulated data sets) is greater than the spread of Sy.x. In 1–2% of the simulated experiments, the RSDR is quite low because two-thirds of the points are very close to the curve. In these cases, the t ratios are high and the P values are low, resulting in more outliers removed.

What happens if the data set is contaminated with lots of outliers? Since we define the RSDR based on the position of the residual at the 68^th ^percentile, this method will work well with up to 32 percent outliers. In fact, our implementation of the outlier removal method only tests the largest 30% residuals. If you have more outliers than that, this method won't be useful. But if more than 30% of your data are outliers, no data analysis method is going to be very helpful.

### When should you use automatic outlier removal?

#### Is it 'cheating' to remove outliers?

Some people feel that removing outliers is 'cheating'. It can be viewed that way when outliers are removed in an *ad hoc *manner, especially when you remove only outliers that get in the way of obtaining results you like. But leaving outliers in the data you analyze is also 'cheating', as it can lead to invalid results.

The ROUT method is automatic. The decision of whether or not to remove an outlier is based only on the distance of the point from the robust best-fit curve.

Here is a Bayesian way to think about this approach. When your experiment has a value flagged as an outlier, there are two possibilities. One possibility is that a coincidence occurred, the kind of coincidence that happens in 1–3% of experiments even if the entire scatter is Gaussian. The other possibility is that a 'bad' point got included in your data. Which possibility is more likely? It depends on your experimental system. If it seems reasonable to assume that your experimental system generates one or more 'bad' points in a few percent of experiments, then it makes sense to eliminate the point as an outlier. It is more likely to be a 'bad' point than a 'good' point that just happened to be far from the curve. If your system is very pure and controlled, so 'bad' points occur in less than 1% of experiments, then it is more likely that the point is far from the curve due to chance (and not mistake) and you should leave it in. Alternatively in that case, you could set Q to a lower value in order to only detect outliers that are much further away.

#### Don't eliminate outliers unless you are sure you are fitting the right model

**Figure 9:**
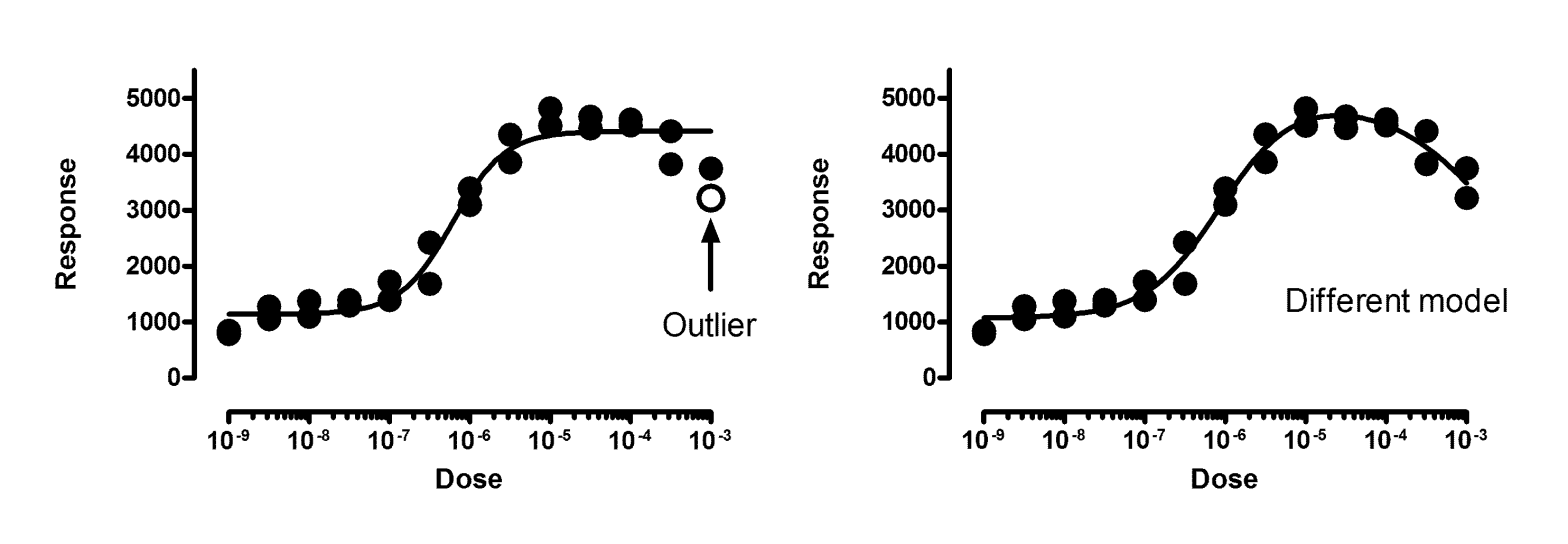
**Don't eliminate outliers unless you are sure you are fitting the correct model**. The left panel shows the data fit with our method to a sigmoid dose response curve. One of the points is declared to be an outlier and removed. The right panel shows the data fit to an alternative model with a biphasic dose-response curve. When fit with this model, none of the points are outliers.

Figure 9 shows the same data fit two ways. The left panel shows the data fit to a standard dose response curve. In this figure, one of the points is a significant outlier (with Q set to 1%). That means that by chance alone (assuming Gaussian scatter) you'd find a point this far from the curve in only 1% of experiments. But this interpretation assumes that you've chosen the correct model. The right panel shows the data fit to an alternative 'bell-shaped' dose-response model, where high doses evoke a smaller response than does a moderate dose. The data fit this model very well, with no outliers detected (or even suspected).

This example points out that outlier elimination is only appropriate when you are sure that you are fitting the correct model.

#### Outlier elimination is misleading when data points are not independent

**Figure 10:**
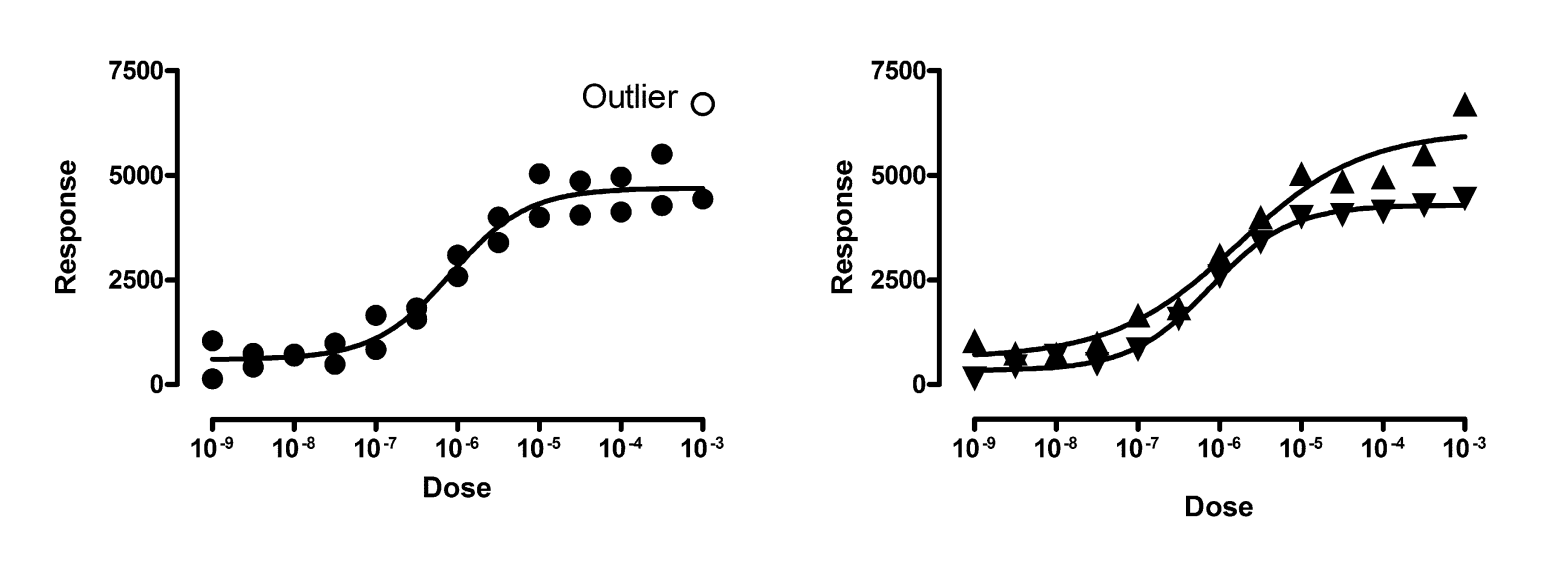
**Don't eliminate outliers when the data are not independent**. The left panel treats the values as unmatched duplicates, and one point is found to be an outlier. The right panel shows that, in fact, the graph superimposes two different curves from two distinct subjects, and none of the points are outliers.

The left panel of Figure 10 show data fit to a dose-response model using outlier elimination with Q set to 1%. One point (in the upper right) is detected as an outlier. The right panel shows that the data really come from two different experiments. Both the lower and upper plateaus of the second experiment (shown with upward pointing triangles) are higher than those in the first experiment (downward pointing triangles). Because these are two different experiments, the assumption of independence was violated in the analysis in the left panel. When we fit each experimental run separately, no outliers are detected, not even if we increase Q from 1% to 10%.

#### Outlier elimination is misleading when you haven't chosen weighting factors appropriately

**Figure 11:**
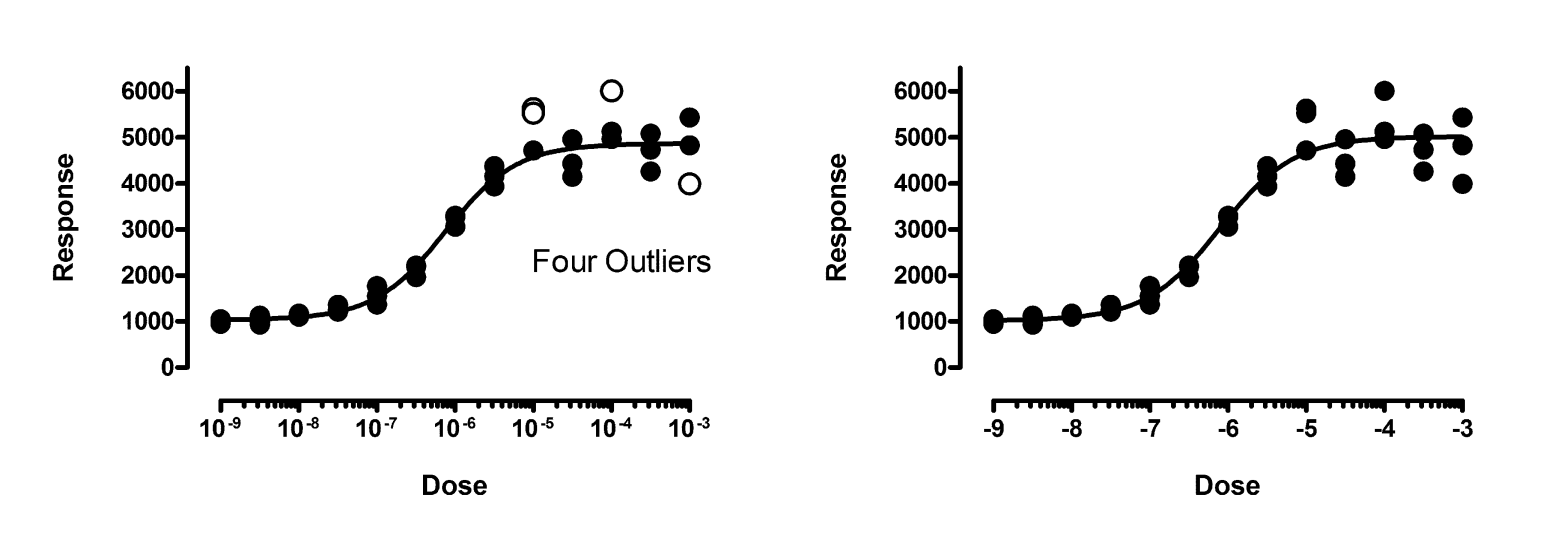
**Don't use outlier elimination if you don't use weighting correctly**. The graph shows data simulated with Gaussian scatter with a standard deviation equal to 10% of the Y value. The left panel shows our method used incorrectly, without adjusting for the fact that the scatter increases as Y increases. Four outliers are identified, all incorrectly. The right panel shows the correct analysis, where weighted residuals are used to define outliers, and no outliers are found.

The left panel of Figure 11 shows data fit to a dose-response model. Four outliers were identified with Q set to 1% (two are almost superimposed). But note that the values with larger responses (Y values) also, on average, are further from the curve. This makes least-squares regression inappropriate. To account for the fact that the SD of the residuals is proportional to the height of the curve, we need to use weighted regression. The right panel shows the same data fit to the same dose-response model, but minimizing sum of the squares of the distance of the point from the curve divided by the height of the curve, using relative weighting. Now no outliers are identified. Using the wrong weighting method created false outliers.

#### Outlier elimination is misleading when a value can be an outlier due to biological variation rather than experimental error

Nonlinear regression is usually used with experimental data, where X is a variable like time or concentration or some other variable you manipulate in the experiment. Since all the scatter is due to experimental error, it can make sense to eliminate any extreme outlier since it is almost certainly the result of an experimental mistake.

In other situations, each data point can represent a different individual. In this case, an outlier may not be due to experimental mistakes, but rather be the result of biological variation, or differences in some other variable that is not included in your model. Here, the presence of the outlier may be the most interesting finding in the study. While our outlier method might prove useful to flag an outlier in this situation, it would be a mistake to automatically exclude such outliers without further examination (perhaps repeating the measurement in those subjects).

### Future directions

We foresee several extensions to our ROUT method. We plan to extend the method so it can be used when comparing alternative models. We also plan to investigate whether the procedure can be simplified when identifying possible outliers from a set of values assumed to be sampled from a Gaussian distribution. Another extension might be to base outlier detection on variations of the FDR method [7-9].

## Conclusion

We describe a new method for identifying outliers when fitting data with nonlinear regression. This method combines robust regression and outlier removal, and so we call it the ROUT method. Analyses of simulated data demonstrate that this method identifies outliers from nonlinear curve fits with reasonable power and few false positives. We have implemented this method in version 5 of GraphPad Prism, which we anticipate releasing in 2006 ().

## Methods

### Calculating the RSDR

Our method requires calculation of the robust standard deviation of the residuals (RSDR). During each iteration, this value scales the residuals and so adjusts the degree of robustness (as shown in Equation 8 below). As the curve gets closer to the points, the RSDR decreases, and so the fitting method becomes more robust. After the robust fitting is complete, we also use the RSDR to evaluate which points are far enough from the curve to be eliminated as outliers (as shown in Equation 18).

In a Gaussian distribution, you expect 68.27% of the values to lie within one standard deviation of the mean. To find a robust standard deviation, therefore, therefore we take the absolute value of the residuals and find the 68.27 percentile (using proportional interpolation between values that bracket the 68.27 percentile). We call this value P68.

We used simulations (of data with Gaussian scatter) to compare the value of P68 with the standard error of the fit (called S_y.x _or S_e_). With low sample sizes, we found that the P68 was too low, and that Equation 1 computes a much more accurate robust standard deviation. In this equation, N is the number of data points and K is the number of parameters fit by nonlinear regression, so the correction factor only matters when N is small.

RSDR=P68NN−K     1
 MathType@MTEF@5@5@+=feaafiart1ev1aaatCvAUfKttLearuWrP9MDH5MBPbIqV92AaeXatLxBI9gBaebbnrfifHhDYfgasaacH8akY=wiFfYdH8Gipec8Eeeu0xXdbba9frFj0=OqFfea0dXdd9vqai=hGuQ8kuc9pgc9s8qqaq=dirpe0xb9q8qiLsFr0=vr0=vr0dc8meaabaqaciaacaGaaeqabaqabeGadaaakeaacqWGsbGucqWGtbWucqWGebarcqWGsbGucqGH9aqpcqWGqbaucqaI2aGncqaI4aaodaWcaaqaaiabd6eaobqaaiabd6eaojabgkHiTiabdUealbaacaWLjaGaaCzcaiabigdaXaaa@3C07@

We simulated several kinds of curves, with different numbers of data points and parameters, always analyzing 5000 simulated data sets with Gaussian scatter. We found that average RSDR computed this way is always within a few percent of the average S_y.x _from least squares fit. The discrepancies between the average values of RSDR and S_y.x _were much smaller (<30%) than the standard deviation of the S_y.x _values.

We have not seen the P68 used before to compute a robust standard deviation. The most common method is to compute the median absolute deviation (MAD) of the points from the curve, and then compute a robust standard deviation by dividing the MAD by 0.6745 (because 50% of a Gaussian distribution lies within 0.6745 standard deviations of the mean). We found that the RSDR values calculated this way vary more between simulations than do the RSDR values computed from the P68. This makes sense because the P68 takes into account more of the data. The breakdown point of the MAD is 50%, which means you can change 50% of the values (starting from the largest) to an arbitrarily high value and not change the MAD. The breakdown point of p68 is 32%. Since our method is designed to detect a small percentage of outliers, a breakdown point of 32% is quite acceptable, and using the P68 rather than the MAD gives us a slightly more accurate assessment of the standard deviation.

At low sample sizes, this MAD method underestimates the sample standard deviation [10]. Croux and Rousseeuw report some empirical correction factors that can be used so the expected value of the MAD/0.6745 equals the sample standard deviation of the values [11]. But these correction factors are not quite right for our situation, where we often are fitting multiple parameters (K>1) and so need to make our RSDR match not the SD of the residuals (the square root of the sum-of-squares divided by N-1), but rather the Sy.x of the fit (the square root of the sum-of-squares divided by N-K). Since the factors proposed by Croux and Rousseeuw were derived empirically, with no theoretical basis, we chose to use our own correction factors as shown above in Equation 1.

Rousseeuw and Verboven reported on a method for computing a robust standard deviation from small samples [12]. We tried using their equation 27, and found that it did not result in a more accurate or less variable RSDR, and did not remove the need for the correction factor for small sample sizes.

### Robust merit function

#### Why is ordinary regression called 'least squares'?

Before explaining how the robust regression works, let's review ordinary regression, and explain why it has the goal of minimizing the sum of square of the residuals.

**Figure 12:**
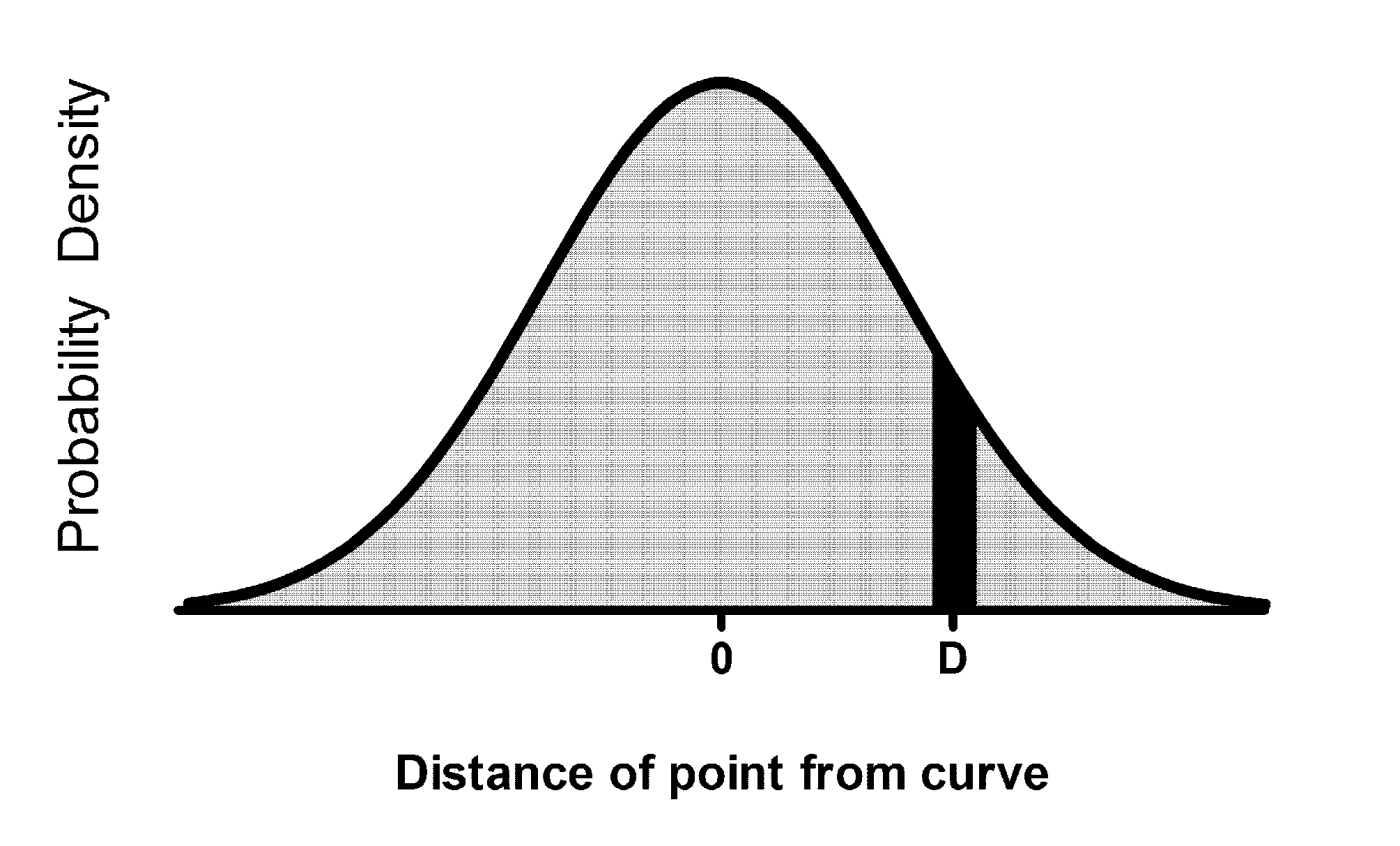
**A Gaussian probability density curve**. The ratio of the black area to the entire area under the curve is the probability that a value selected from this Gaussian distribution will have a value of D plus or minus ΔD.

First let's back up and ask: Given the assumption that scatter is Gaussian, what is the chance that a point will be a certain distance D from the curve? That can't really be answered, but a related question can be answered: What is the chance that the distance between point and curve will be a certain distance D plus or minus ΔD? The area under the entire probability density curve (Figure 12) represents all possible values of D, so the area under a certain part of the curve (for example the portion shown in black in Figure 12) represents the probability that D will be within that range.

So the answer to our question is the ratio of the area in the dark area (in Figure 12) divided by the total area (which we'll call A). The area in the dark area, given that ΔD is tiny, is the height of the Gaussian distribution at D multiplied by 2ΔD, where ΔD is an arbitrary small number.

Probability=2ΔD⋅e−12D2A     2
 MathType@MTEF@5@5@+=feaafiart1ev1aaatCvAUfKttLearuWrP9MDH5MBPbIqV92AaeXatLxBI9gBaebbnrfifHhDYfgasaacH8akY=wiFfYdH8Gipec8Eeeu0xXdbba9frFj0=OqFfea0dXdd9vqai=hGuQ8kuc9pgc9s8qqaq=dirpe0xb9q8qiLsFr0=vr0=vr0dc8meaabaqaciaacaGaaeqabaqabeGadaaakeaacqqGqbaucqqGYbGCcqqGVbWBcqqGIbGycqqGHbqycqqGIbGycqqGPbqAcqqGSbaBcqqGPbqAcqqG0baDcqqG5bqEcqGH9aqpdaWcaaqaaiabikdaYiabfs5aejabdseaejabgwSixlabdwgaLnaaCaaaleqabaGaeyOeI0YaaSaaaeaacqaIXaqmaeaacqaIYaGmaaGaemiraq0aaWbaaWqabeaacqaIYaGmaaaaaaGcbaGaemyqaeeaaiaaxMaacaWLjaGaeGOmaidaaa@4C1F@

That equation is for one data point. Now let's generalize to the entire set of data points. What is the chance that a particular set of parameters would have produced all the data points we observed? To answer, we calculate Equation 1 for each data point, and multiply all those probabilities together. The goal of curve fitting is to alter the values of the parameters to find the curve that maximizes that product of probabilities.

The simplest way to maximize the product of a set of numbers is to maximize the sum of their logarithms. That is equivalent to minimizing the sum of the negative of the logarithms. So we want to minimize this merit function:

Gaussian Merit=−ln⁡[∑i=1N(2⋅ΔD⋅e−12D2A)]=[∑i=1N(D2)]A−NAln⁡(2⋅ΔD)     3
 MathType@MTEF@5@5@+=feaafiart1ev1aaatCvAUfKttLearuWrP9MDH5MBPbIqV92AaeXatLxBI9gBaebbnrfifHhDYfgasaacH8akY=wiFfYdH8Gipec8Eeeu0xXdbba9frFj0=OqFfea0dXdd9vqai=hGuQ8kuc9pgc9s8qqaq=dirpe0xb9q8qiLsFr0=vr0=vr0dc8meaabaqaciaacaGaaeqabaqabeGadaaakeaafaqadeGabaaabaGaee4raCKaeeyyaeMaeeyDauNaee4CamNaee4CamNaeeyAaKMaeeyyaeMaeeOBa4MaeeiiaaIaeeyta0KaeeyzauMaeeOCaiNaeeyAaKMaeeiDaqNaeyypa0JaeyOeI0IagiiBaWMaeiOBa42aamWaaeaadaaeWbqaamaabmaabaWaaSaaaeaacqaIYaGmcqGHflY1cqqHuoarcqWGebarcqGHflY1cqWGLbqzdaahaaWcbeqaaiabgkHiTmaalaaabaGaeGymaedabaGaeGOmaidaaiabdseaenaaCaaameqabaGaeGOmaidaaaaaaOqaaiabdgeabbaaaiaawIcacaGLPaaaaSqaaiabdMgaPjabg2da9iabigdaXaqaaiabd6eaobqdcqGHris5aaGccaGLBbGaayzxaaaabaGaeyypa0ZaaSaaaeaadaWadaqaamaaqahabaWaaeWaaeaacqWGebardaahaaWcbeqaaiabikdaYaaaaOGaayjkaiaawMcaaaWcbaGaemyAaKMaeyypa0JaeGymaedabaGaemOta4eaniabggHiLdaakiaawUfacaGLDbaaaeaacqWGbbqqaaGaeyOeI0YaaSaaaeaacqWGobGtaeaacqWGbbqqaaGagiiBaWMaeiOBa4MaeiikaGIaeGOmaiJaeyyXICTaeuiLdqKaemiraqKaeiykaKcaaiaaxMaacaWLjaGaeG4mamdaaa@7BA5@

When we change the values of the parameters, the curve changes, so the values of D change. The value ΔD is an arbitrary very small number, and the values of N and A don't depend on the values of the parameters that define the curve. Since the term on the right is constant, it can be ignored when adjusting the parameters to minimize the merit function. We can also remove the constant A from the left side. So to find the values of the parameters that have the maximum likelihood of being correct, we therefore minimize this simpler merit function:

Gaussian Merit=∑i=1N(D2)     4
 MathType@MTEF@5@5@+=feaafiart1ev1aaatCvAUfKttLearuWrP9MDH5MBPbIqV92AaeXatLxBI9gBaebbnrfifHhDYfgasaacH8akY=wiFfYdH8Gipec8Eeeu0xXdbba9frFj0=OqFfea0dXdd9vqai=hGuQ8kuc9pgc9s8qqaq=dirpe0xb9q8qiLsFr0=vr0=vr0dc8meaabaqaciaacaGaaeqabaqabeGadaaakeaacqqGhbWrcqqGHbqycqqG1bqDcqqGZbWCcqqGZbWCcqqGPbqAcqqGHbqycqqGUbGBcqqGGaaicqqGnbqtcqqGLbqzcqqGYbGCcqqGPbqAcqqG0baDcqGH9aqpdaaeWbqaamaabmaabaGaemiraq0aaWbaaSqabeaacqaIYaGmaaaakiaawIcacaGLPaaaaSqaaiabdMgaPjabg2da9iabigdaXaqaaiabd6eaobqdcqGHris5aOGaaCzcaiaaxMaacqaI0aanaaa@4C8B@

Equation 4 shows the familiar goal of regression – to minimize the sums of squares of the distances (D) of the curve from the points.

#### Merit function for robust regression

To adjust this equation for robust regression, we need to use the equation for the Lorentzian, rather than Gaussian distribution. The height of the Lorentzian probability density function at a distance D from the center is

Height=11+0.5D2     5
 MathType@MTEF@5@5@+=feaafiart1ev1aaatCvAUfKttLearuWrP9MDH5MBPbIqV92AaeXatLxBI9gBaebbnrfifHhDYfgasaacH8akY=wiFfYdH8Gipec8Eeeu0xXdbba9frFj0=OqFfea0dXdd9vqai=hGuQ8kuc9pgc9s8qqaq=dirpe0xb9q8qiLsFr0=vr0=vr0dc8meaabaqaciaacaGaaeqabaqabeGadaaakeaacqqGibascqqGLbqzcqqGPbqAcqqGNbWzcqqGObaAcqqG0baDcqGH9aqpdaWcaaqaaiabigdaXaqaaiabigdaXiabgUcaRiabicdaWiabc6caUiabiwda1iabdseaenaaCaaaleqabaGaeGOmaidaaaaakiaaxMaacaWLjaGaeGynaudaaa@3FA0@

So the probability (corresponding to equation 1) that a point will be a distance D (plus or minus a small value ΔD) is

Probability=2ΔDA(1+0.5D2)     6
 MathType@MTEF@5@5@+=feaafiart1ev1aaatCvAUfKttLearuWrP9MDH5MBPbIqV92AaeXatLxBI9gBaebbnrfifHhDYfgasaacH8akY=wiFfYdH8Gipec8Eeeu0xXdbba9frFj0=OqFfea0dXdd9vqai=hGuQ8kuc9pgc9s8qqaq=dirpe0xb9q8qiLsFr0=vr0=vr0dc8meaabaqaciaacaGaaeqabaqabeGadaaakeaacqqGqbaucqqGYbGCcqqGVbWBcqqGIbGycqqGHbqycqqGIbGycqqGPbqAcqqGSbaBcqqGPbqAcqqG0baDcqqG5bqEcqGH9aqpdaWcaaqaaiabikdaYiabfs5aejabdseaebqaaiabdgeabjabcIcaOiabigdaXiabgUcaRiabicdaWiabc6caUiabiwda1iabdseaenaaCaaaleqabaGaeGOmaidaaOGaeiykaKcaaiaaxMaacaWLjaGaeGOnaydaaa@4BCB@

We want to maximize the product of these probabilities, which is the same as minimizing the sum of the negative logarithms. Omitting the constant terms, this means we want to minimize this merit function:

Lorentzian Merit=∑iln⁡[1+D22]     7
 MathType@MTEF@5@5@+=feaafiart1ev1aaatCvAUfKttLearuWrP9MDH5MBPbIqV92AaeXatLxBI9gBaebbnrfifHhDYfgasaacH8akY=wiFfYdH8Gipec8Eeeu0xXdbba9frFj0=OqFfea0dXdd9vqai=hGuQ8kuc9pgc9s8qqaq=dirpe0xb9q8qiLsFr0=vr0=vr0dc8meaabaqaciaacaGaaeqabaqabeGadaaakeaacqqGmbatcqqGVbWBcqqGYbGCcqqGLbqzcqqGUbGBcqqG0baDcqqG6bGEcqqGPbqAcqqGHbqycqqGUbGBcqqGGaaicqqGnbqtcqqGLbqzcqqGYbGCcqqGPbqAcqqG0baDcqGH9aqpdaaeqbqaaiGbcYgaSjabc6gaUbWcbaGaemyAaKgabeqdcqGHris5aOWaamWaaeaacqaIXaqmcqGHRaWkdaWcaaqaaiabdseaenaaCaaaleqabaGaeGOmaidaaaGcbaGaeGOmaidaaaGaay5waiaaw2faaiaaxMaacaWLjaGaeG4naCdaaa@523E@

Equation 7 seems analogous to Equation 4, but in fact Equation 7 isn't useful as written. The problem is that if you change the units of Y, different parameter values (and thus a different curve) will minimize that merit score. That is not acceptable. As pointed out by Draper and Smith [13] (pages 568–572) and Seber and Wild [14], the distances D must be divided by a scale factor expressed in the same units as the Y values of the data points and curve. We chose to scale the Y values to the RSDR, explained above.

The goal of our robust nonlinear regression method, therefore, is to minimize:

Lorentzian Merit=∑ln⁡(1+(DRSDR)2)     8
 MathType@MTEF@5@5@+=feaafiart1ev1aaatCvAUfKttLearuWrP9MDH5MBPbIqV92AaeXatLxBI9gBaebbnrfifHhDYfgasaacH8akY=wiFfYdH8Gipec8Eeeu0xXdbba9frFj0=OqFfea0dXdd9vqai=hGuQ8kuc9pgc9s8qqaq=dirpe0xb9q8qiLsFr0=vr0=vr0dc8meaabaqaciaacaGaaeqabaqabeGadaaakeaacqqGmbatcqqGVbWBcqqGYbGCcqqGLbqzcqqGUbGBcqqG0baDcqqG6bGEcqqGPbqAcqqGHbqycqqGUbGBcqqGGaaicqqGnbqtcqqGLbqzcqqGYbGCcqqGPbqAcqqG0baDcqGH9aqpdaaeabqaaiGbcYgaSjabc6gaUnaabmaabaGaeGymaeJaey4kaSYaaeWaaeaadaWcaaqaaiabdseaebqaaiabdkfasjabdofatjabdseaejabdkfasbaaaiaawIcacaGLPaaadaahaaWcbeqaaiabikdaYaaaaOGaayjkaiaawMcaaaWcbeqab0GaeyyeIuoakiaaxMaacaWLjaGaeGioaGdaaa@559E@

#### Why does assuming a Lorentzian distribution of residuals make the fit robust?

**Figure 13:**
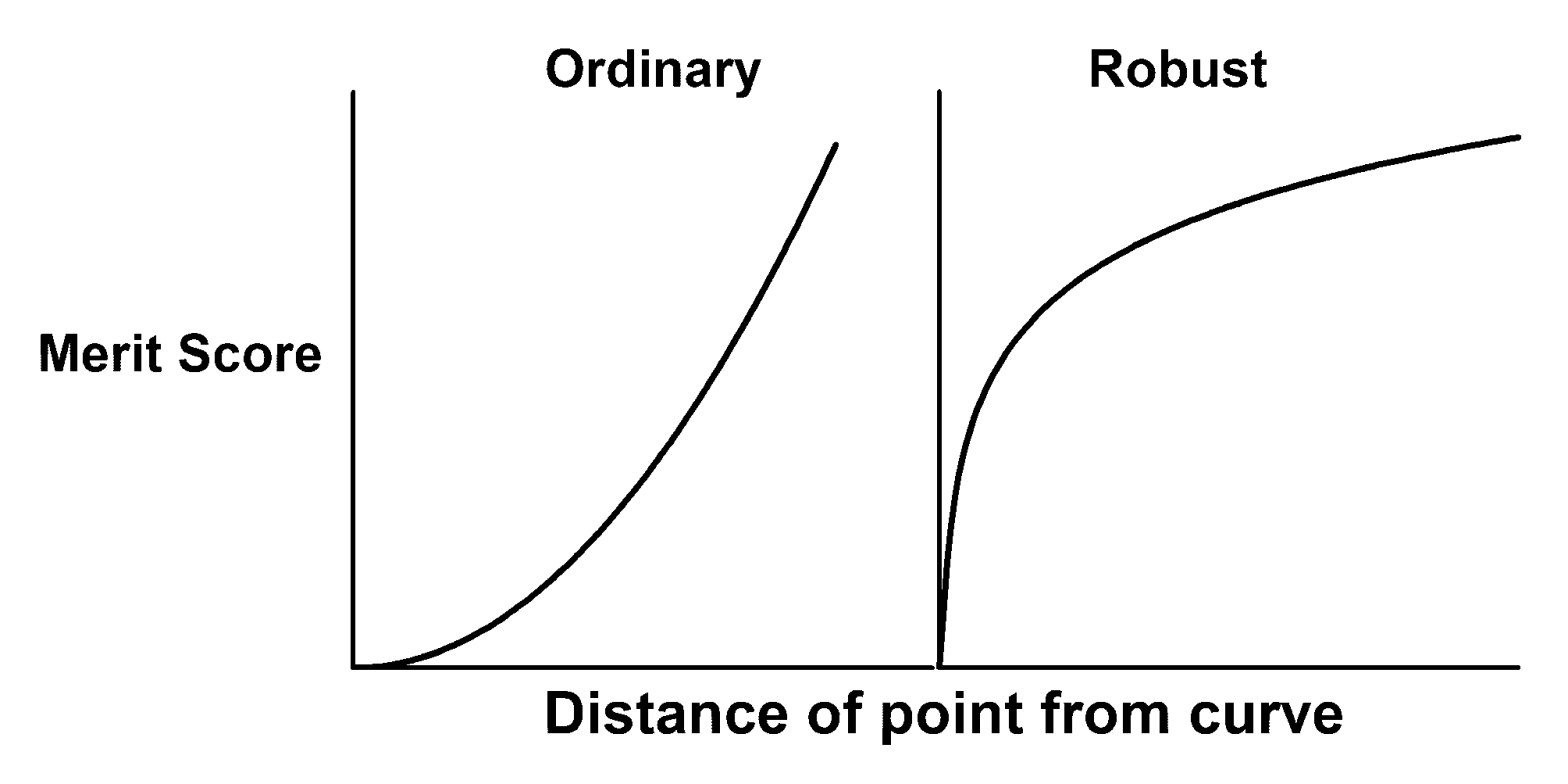
**Why assuming a Lorentzian distribution of residuals makes the fitting process robust**. The graph shows the contribution of a point to the merit score for Gaussian (left) and Lorentzian (right) as a function of the distance of a point from the curve. The goal of curve fitting is to minimize the merit score. The curve in the right panel starts to level off. This means that moving the curve a bit closer to, or further from, a point that is already far from the curve won't change the merit score very much. This is the definition of a robust fitting method. In contrast, the curve on the left does not level off, so points far from the curve have a huge impact on least squares fitting.

Figure 13 shows how merit score varies with the distance of the point from the curve. Recall that the goal is to minimize this merit function. At first glance, you may be puzzled by the right panel. Points that are far from the curve (right side of the graph) have high values for the merit score. How can the method be robust?

The influence of a point on regression is defined as the change in goodness-of-fit score when the curve is moved a bit closer to, or further from, that point. Consider first the left panel, showing the least-squares regression. The merit score changes substantially if the curve is moved closer to, or further from, points far from the curve. Thus points far from the curve have a huge influence. Now compare to the right panel, showing the robust regression. The merit score levels off, so does not change much if the curve is moved closer to, or further from, points that are already far from the curve. This is the definition of a robust method.

#### Why is the method adaptive?

**Figure 14:**
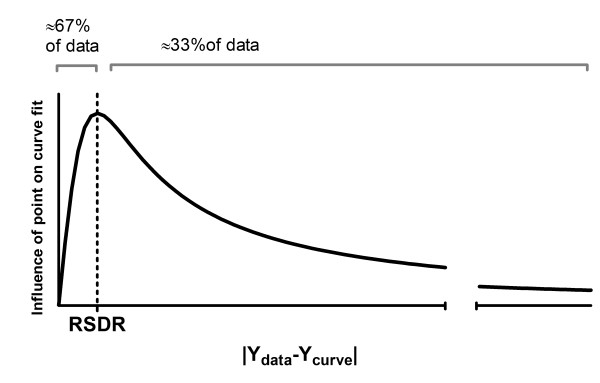
**The influence curve of robust fitting**. This curve is the derivative of the curve shown in the right panel of Figure 13. The influence peaks for points whose distance from the curve equals the robust standard deviation of the residuals (RSDR). The RSDR is recomputed every iteration. This means that about two-thirds of the points get about the same influence they would have had with least-squares regression.

Figure 14 shows the influence of a data point as a function of its distance from the curve. We define the influence as the derivative of the goodness-of-fit merit score (defined in Equation 8) with respect to the distance of the point from the curve. The RSDR is the peak of the influence curve. About two thirds (68.27%) of the data points are closer to the curve than the RSDR. For these points, robust regression is very similar to least-squares regression, with influence almost proportional to distance (the left portion of the influence curve, up to its peak, is close to a straight line). The one-third of points furthest from the curve (to the right on this graph) get less influence with robust fitting than they would with least squares.

Figure 14 also explains our choice to divide by the RSDR in Equation 8. The choice of the denominator in that equation is somewhat arbitrary (so long as it is related to the scale of the Y values). Equation 8 would have been more similar to Equation 7 if we had divided by twice the RSDR. But if we did that, then about 95% of the points would have been to the left the peak of Figure 14, and the method would have been less robust.

Nonlinear regression is an iterative method. It begins with initial estimated values of each parameter, and then gradually changes those parameters to improve the merit score. With robust regression, the RSDR is recomputed with iteration, so the rule that about two-thirds of the data points are to the left of the peak remains true during each iteration. This makes the method adaptive. If the initial values result in a curve far from many data points, the RSDR has a high value. As the curve gets close to the bulk of the data, the RSDR gradually becomes lower, and the method gradually becomes more robust as the curve gets closer to the bulk of the data. This allows the method to work well even in situations where the initial values are not well chosen.

### Minimizing the merit function

#### How to minimize the sum-of-squares in ordinary nonlinear regression

Least-squares regression adjusts the values of one or more parameters (a_0_, a_1_, ... a_k_) to minimize the weighted sum (over all data points) of the squared distance between the y value of the data point (y_1_, y_2_, ... y_N_) and the corresponding y value of the curve. The equations below are written assuming there are two parameters (denoted a_0 _and a_1_; these might correspond to V_max _and K_m _when fitting enzyme kinetics data). The Y value for the curve is denoted by y^
 MathType@MTEF@5@5@+=feaafiart1ev1aaatCvAUfKttLearuWrP9MDH5MBPbIqV92AaeXatLxBI9gBaebbnrfifHhDYfgasaacH8akY=wiFfYdH8Gipec8Eeeu0xXdbba9frFj0=OqFfea0dXdd9vqai=hGuQ8kuc9pgc9s8qqaq=dirpe0xb9q8qiLsFr0=vr0=vr0dc8meaabaqaciaacaGaaeqabaqabeGadaaakeaacuWG5bqEgaqcaaaa@2E37@(*x*_*i*_, *a*_0_, *a*_1_) meaning the predicted value of y given particular values for x and the two parameters.

Equation 9 restates the merit function for ordinary regression and is equivalent to Equation 4.

Gaussain Merit=∑i[yi−y^(xi,a0,a1)]2     9
 MathType@MTEF@5@5@+=feaafiart1ev1aaatCvAUfKttLearuWrP9MDH5MBPbIqV92AaeXatLxBI9gBaebbnrfifHhDYfgasaacH8akY=wiFfYdH8Gipec8Eeeu0xXdbba9frFj0=OqFfea0dXdd9vqai=hGuQ8kuc9pgc9s8qqaq=dirpe0xb9q8qiLsFr0=vr0=vr0dc8meaabaqaciaacaGaaeqabaqabeGadaaakeaacqqGhbWrcqqGHbqycqqG1bqDcqqGZbWCcqqGZbWCcqqGHbqycqqGPbqAcqqGUbGBcqqGGaaicqqGnbqtcqqGLbqzcqqGYbGCcqqGPbqAcqqG0baDcqGH9aqpdaaeqbqaaiabcUfaBjabdMha5naaBaaaleaacqWGPbqAaeqaaOGaeyOeI0IafmyEaKNbaKaacqGGOaakcqWG4baEdaWgaaWcbaGaemyAaKgabeaakiabcYcaSiabdggaHnaaBaaaleaacqaIWaamaeqaaOGaeiilaWIaemyyae2aaSbaaSqaaiabigdaXaqabaGccqGGPaqkcqGGDbqxdaahaaWcbeqaaiabikdaYaaaaeaacqWGPbqAaeqaniabggHiLdGccaWLjaGaaCzcaiabiMda5aaa@5A0C@

Nonlinear regression requires calculating the Hessian matrix (also called the design matrix). This is square, with the number of rows and columns equal to K, the number of parameters that will be fit by regression. Each diagonal element in the matrix is the square of the partial derivative of the merit function with respect to the parameter defined by the row or column of the matrix. Each non-diagonal element in the matrix is the product of the partial derivatives of the merit function with respect to the parameters defined by the row and column of the matrix.

First define the first derivative of the merit function with respect to a parameter a_0_.

Gaussian Merita0′=∑i−2[yi−y^(xi,a0,a1)][∂∂a0y^(xi,a0,a1)]     10
 MathType@MTEF@5@5@+=feaafiart1ev1aaatCvAUfKttLearuWrP9MDH5MBPbIqV92AaeXatLxBI9gBaebbnrfifHhDYfgasaacH8akY=wiFfYdH8Gipec8Eeeu0xXdbba9frFj0=OqFfea0dXdd9vqai=hGuQ8kuc9pgc9s8qqaq=dirpe0xb9q8qiLsFr0=vr0=vr0dc8meaabaqaciaacaGaaeqabaqabeGadaaakeaacqqGhbWrcqqGHbqycqqG1bqDcqqGZbWCcqqGZbWCcqqGPbqAcqqGHbqycqqGUbGBcqqGGaaicqqGnbqtcqqGLbqzcqqGYbGCcqqGPbqAcuqG0baDgaqbamaaBaaaleaaieGacqWFHbqydaWgaaadbaGaeeimaadabeaaaSqabaGccqGH9aqpdaaeqbqaaiabgkHiTiabikdaYiabcUfaBjabdMha5naaBaaaleaacqWGPbqAaeqaaOGaeyOeI0IafmyEaKNbaKaacqGGOaakcqWG4baEdaWgaaWcbaGaemyAaKgabeaakiabcYcaSiabdggaHnaaBaaaleaacqaIWaamaeqaaOGaeiilaWIaemyyae2aaSbaaSqaaiabigdaXaqabaGccqGGPaqkcqGGDbqxdaWadaqaamaalaaabaacciGae4NaIylabaGae4NaIyRaemyyae2aaSbaaSqaaiabicdaWaqabaaaaOGafmyEaKNbaKaacqGGOaakcqWG4baEdaWgaaWcbaGaemyAaKgabeaakiabcYcaSiabdggaHnaaBaaaleaacqaIWaamaeqaaOGaeiilaWIaemyyae2aaSbaaSqaaiabigdaXaqabaGccqGGPaqkaiaawUfacaGLDbaaaSqaaiabdMgaPbqab0GaeyyeIuoakiaaxMaacaWLjaGaeGymaeJaeGimaadaaa@7288@

Now define the second derivative with respect to two parameters.

Gaussian Merita0,a1″=∑i(2[∂∂a0y^(xi,a0,a1)][∂∂a1y^(xi,a0,a1)]−{2[yi−y^(xi,a0,a1)](∂2∂a0a1y^(xi,a0,a1)})     11
 MathType@MTEF@5@5@+=feaafiart1ev1aaatCvAUfKttLearuWrP9MDH5MBPbIqV92AaeXatLxBI9gBaebbnrfifHhDYfgasaacH8akY=wiFfYdH8Gipec8Eeeu0xXdbba9frFj0=OqFfea0dXdd9vqai=hGuQ8kuc9pgc9s8qqaq=dirpe0xb9q8qiLsFr0=vr0=vr0dc8meaabaqaciaacaGaaeqabaqabeGadaaakeaacqqGhbWrcqqGHbqycqqG1bqDcqqGZbWCcqqGZbWCcqqGPbqAcqqGHbqycqqGUbGBcqqGGaaicqqGnbqtcqqGLbqzcqqGYbGCcqqGPbqAcuqG0baDgaGbamaaBaaaleaacqqGHbqydaWgaaadbaGaeGimaadabeaaliabcYcaSiabbggaHnaaBaaameaacqaIXaqmaeqaaaWcbeaakiabg2da9maaqafabaWaaeWaaeaafaqaaeGabaaabaGaeGOmaiZaamWaaeaadaWcaaqaaGGaciab=jGi2cqaaiab=jGi2kabdggaHnaaBaaaleaacqaIWaamaeqaaaaakiqbdMha5zaajaGaeiikaGIaemiEaG3aaSbaaSqaaiabdMgaPbqabaGccqGGSaalcqWGHbqydaWgaaWcbaGaeGimaadabeaakiabcYcaSiabdggaHnaaBaaaleaacqaIXaqmaeqaaOGaeiykaKcacaGLBbGaayzxaaWaamWaaeaadaWcaaqaaiabgkGi2cqaaiab=jGi2kabdggaHnaaBaaaleaacqaIXaqmaeqaaaaakiqbdMha5zaajaGaeiikaGIaemiEaG3aaSbaaSqaaiabdMgaPbqabaGccqGGSaalcqWGHbqydaWgaaWcbaGaeGimaadabeaakiabcYcaSiabdggaHnaaBaaaleaacqaIXaqmaeqaaOGaeiykaKcacaGLBbGaayzxaaaabaGaeyOeI0YaaiWaaeaacqaIYaGmcqGGBbWwcqWG5bqEdaWgaaWcbaGaemyAaKgabeaakiabgkHiTiqbdMha5zaajaGaeiikaGIaemiEaG3aaSbaaSqaaiabdMgaPbqabaGccqGGSaalcqWGHbqydaWgaaWcbaGaeGimaadabeaakiabcYcaSiabdggaHnaaBaaaleaacqaIXaqmaeqaaOGaeiykaKIaeiyxa01aaeWaaeaadaWcaaqaaiab=jGi2oaaCaaaleqabaGaeGOmaidaaaGcbaGae8NaIyRaemyyae2aaSbaaSqaaiabicdaWaqabaGccqWGHbqydaWgaaWcbaGaeGymaedabeaaaaGccuWG5bqEgaqcaiabcIcaOiabdIha4naaBaaaleaacqWGPbqAaeqaaOGaeiilaWIaemyyae2aaSbaaSqaaiabicdaWaqabaGccqGGSaalcqWGHbqydaWgaaWcbaGaeGymaedabeaaaOGaayjkaiaawMcaaaGaay5Eaiaaw2haaaaaaiaawIcacaGLPaaaaSqaaiabdMgaPbqab0GaeyyeIuoakiaaxMaacaWLjaGaeGymaeJaeGymaedaaa@A520@

The term in {braces}, when summed over all data points, will be close to zero because the first part of that term [in brackets] is the distance of the point from the curve, and you expect about half of these distances to be positive and half to be negative, making the resulting terms cancel out (approximately). Most implementations of nonlinear regression make the assumption that the term in brackets will be negligible, and that any attempt to calculate it might be counterproductive due to round off errors. Therefore, the Hessian matrix used in least-squares nonlinear regression programs is:

Gaussian Merita0,a1″=∑i2[∂∂a0y^(xi,a0,a1)][∂∂a1y^(xi,a0,a1)]     12
 MathType@MTEF@5@5@+=feaafiart1ev1aaatCvAUfKttLearuWrP9MDH5MBPbIqV92AaeXatLxBI9gBaebbnrfifHhDYfgasaacH8akY=wiFfYdH8Gipec8Eeeu0xXdbba9frFj0=OqFfea0dXdd9vqai=hGuQ8kuc9pgc9s8qqaq=dirpe0xb9q8qiLsFr0=vr0=vr0dc8meaabaqaciaacaGaaeqabaqabeGadaaakeaacqqGhbWrcqqGHbqycqqG1bqDcqqGZbWCcqqGZbWCcqqGPbqAcqqGHbqycqqGUbGBcqqGGaaicqqGnbqtcqqGLbqzcqqGYbGCcqqGPbqAcuqG0baDgaGbamaaBaaaleaacqqGHbqydaWgaaadbaGaeGimaadabeaaliabcYcaSiabbggaHnaaBaaameaacqaIXaqmaeqaaaWcbeaakiabg2da9maaqafabaGaeGOmaiZaamWaaeaadaWcaaqaaGGaciab=jGi2cqaaiab=jGi2kabdggaHnaaBaaaleaacqaIWaamaeqaaaaakiqbdMha5zaajaGaeiikaGIaemiEaG3aaSbaaSqaaiabdMgaPbqabaGccqGGSaalcqWGHbqydaWgaaWcbaGaeGimaadabeaakiabcYcaSiabdggaHnaaBaaaleaacqaIXaqmaeqaaOGaeiykaKcacaGLBbGaayzxaaWaamWaaeaadaWcaaqaaiab=jGi2cqaaiab=jGi2kabdggaHnaaBaaaleaacqaIXaqmaeqaaaaakiqbdMha5zaajaGaeiikaGIaemiEaG3aaSbaaSqaaiabdMgaPbqabaGccqGGSaalcqWGHbqydaWgaaWcbaGaeGimaadabeaakiabcYcaSiabdggaHnaaBaaaleaacqaIXaqmaeqaaOGaeiykaKcacaGLBbGaayzxaaaaleaacqWGPbqAaeqaniabggHiLdGccaWLjaGaaCzcaiabigdaXiabikdaYaaa@75A9@

The Marquadt-Levenberg method [15,16] uses the vector of partial derivatives (Equation 10) and the Hessian matrix (Equation 12) to determine how to change the values of the parameters to decrease the merit function. This is repeated until any attempt to change the values of the parameters leads to virtually no change in the merit function. At that point, nonlinear regression is said to have converged, and you have obtained the best-fit values of the parameters.

#### Minimizing the robust merit function

First, let's define RR to be the distance of a point from the curve divided by the robust standard deviation of the residuals.

RR=yi−y^(xi,a0,a1)RSDR     13
 MathType@MTEF@5@5@+=feaafiart1ev1aaatCvAUfKttLearuWrP9MDH5MBPbIqV92AaeXatLxBI9gBaebbnrfifHhDYfgasaacH8akY=wiFfYdH8Gipec8Eeeu0xXdbba9frFj0=OqFfea0dXdd9vqai=hGuQ8kuc9pgc9s8qqaq=dirpe0xb9q8qiLsFr0=vr0=vr0dc8meaabaqaciaacaGaaeqabaqabeGadaaakeaacqWGsbGucqWGsbGucqGH9aqpdaWcaaqaaiabdMha5naaBaaaleaacqWGPbqAaeqaaOGaeyOeI0IafmyEaKNbaKaacqGGOaakcqWG4baEdaWgaaWcbaGaemyAaKgabeaakiabcYcaSiabdggaHnaaBaaaleaacqaIWaamaeqaaOGaeiilaWIaemyyae2aaSbaaSqaaiabigdaXaqabaGccqGGPaqkaeaacqWGsbGucqWGtbWucqWGebarcqWGsbGuaaGaaCzcaiaaxMaacqaIXaqmcqaIZaWmaaa@48BE@

Now we can restate Equation 8 in a simpler form.

Lorentzian Merit=∑iln⁡(1+RR2)     14
 MathType@MTEF@5@5@+=feaafiart1ev1aaatCvAUfKttLearuWrP9MDH5MBPbIqV92AaeXatLxBI9gBaebbnrfifHhDYfgasaacH8akY=wiFfYdH8Gipec8Eeeu0xXdbba9frFj0=OqFfea0dXdd9vqai=hGuQ8kuc9pgc9s8qqaq=dirpe0xb9q8qiLsFr0=vr0=vr0dc8meaabaqaciaacaGaaeqabaqabeGadaaakeaacqqGmbatcqqGVbWBcqqGYbGCcqqGLbqzcqqGUbGBcqqG0baDcqqG6bGEcqqGPbqAcqqGHbqycqqGUbGBcqqGGaaicqqGnbqtcqqGLbqzcqqGYbGCcqqGPbqAcqqG0baDcqGH9aqpdaaeqbqaaiGbcYgaSjabc6gaUbWcbaGaemyAaKgabeqdcqGHris5aOGaeiikaGIaeGymaeJaey4kaSIaemOuaiLaemOuai1aaWbaaSqabeaacqaIYaGmaaGccqGGPaqkcaWLjaGaaCzcaiabigdaXiabisda0aaa@532F@

The derivative of this merit function with respect to a parameter (a_0_) is:

Lorentzian Merita0′=−2RSDR2∑i(yi−y^(xi,a0,a1))[∂∂a0y^(xi,a0,a1)][1+RR2]     15
 MathType@MTEF@5@5@+=feaafiart1ev1aaatCvAUfKttLearuWrP9MDH5MBPbIqV92AaeXatLxBI9gBaebbnrfifHhDYfgasaacH8akY=wiFfYdH8Gipec8Eeeu0xXdbba9frFj0=OqFfea0dXdd9vqai=hGuQ8kuc9pgc9s8qqaq=dirpe0xb9q8qiLsFr0=vr0=vr0dc8meaabaqaciaacaGaaeqabaqabeGadaaakeaacqqGmbatcqqGVbWBcqqGYbGCcqqGLbqzcqqGUbGBcqqG0baDcqqG6bGEcqqGPbqAcqqGHbqycqqGUbGBcqqGGaaicqqGnbqtcqqGLbqzcqqGYbGCcqqGPbqAcuqG0baDgaqbamaaBaaaleaacqqGHbqydaWgaaadbaGaeGimaadabeaaaSqabaGccqGH9aqpdaWcaaqaaiabgkHiTiabikdaYaqaaiabdkfasjabdofatjabdseaejabdkfasnaaCaaaleqabaGaeGOmaidaaaaakmaaqafabaWaaSaaaeaadaqadaqaaiabdMha5naaBaaaleaacqWGPbqAaeqaaOGaeyOeI0IafmyEaKNbaKaacqGGOaakcqWG4baEdaWgaaWcbaGaemyAaKgabeaakiabcYcaSiabdggaHnaaBaaaleaacqaIWaamaeqaaOGaeiilaWIaemyyae2aaSbaaSqaaiabigdaXaqabaGccqGGPaqkaiaawIcacaGLPaaadaWadaqaamaalaaabaacciGae8NaIylabaGae8NaIyRaemyyae2aaSbaaSqaaiabicdaWaqabaaaaOGafmyEaKNbaKaacqGGOaakcqWG4baEdaWgaaWcbaGaemyAaKgabeaakiabcYcaSiabdggaHnaaBaaaleaacqaIWaamaeqaaOGaeiilaWIaemyyae2aaSbaaSqaaiabigdaXaqabaGccqGGPaqkaiaawUfacaGLDbaaaeaadaWadaqaaiabigdaXiabgUcaRiabdkfasjabdkfasnaaCaaaleqabaGaeGOmaidaaaGccaGLBbGaayzxaaaaaaWcbaGaemyAaKgabeqdcqGHris5aOGaaCzcaiaaxMaacqaIXaqmcqaI1aqnaaa@81A9@

The Hessian matrix has three terms. One of those terms includes second derivative terms, assumed to sum to nearly zero, and so is ignored. Another term includes the fourth power of the residuals and RSDR. We found that including this term does not help, and actually leads to slower convergence. So the Hessian matrix we use consists of just one term:

Lorentzian Merita0,a1″=2RSDR2∑i1(1+RR2)[∂∂a0y^(xi,a0,a1)][∂∂a1y^(xi,a0,a1)]     16
 MathType@MTEF@5@5@+=feaafiart1ev1aaatCvAUfKttLearuWrP9MDH5MBPbIqV92AaeXatLxBI9gBaebbnrfifHhDYfgasaacH8akY=wiFfYdH8Gipec8Eeeu0xXdbba9frFj0=OqFfea0dXdd9vqai=hGuQ8kuc9pgc9s8qqaq=dirpe0xb9q8qiLsFr0=vr0=vr0dc8meaabaqaciaacaGaaeqabaqabeGadaaakeaacqqGmbatcqqGVbWBcqqGYbGCcqqGLbqzcqqGUbGBcqqG0baDcqqG6bGEcqqGPbqAcqqGHbqycqqGUbGBcqqGGaaicqqGnbqtcqqGLbqzcqqGYbGCcqqGPbqAcuqG0baDgaGbamaaBaaaleaacqqGHbqydaWgaaadbaGaeGimaadabeaaliabcYcaSiabbggaHnaaBaaameaacqaIXaqmaeqaaaWcbeaakiabg2da9maalaaabaGaeGOmaidabaGaemOuaiLaem4uamLaemiraqKaemOuai1aaWbaaSqabeaacqaIYaGmaaaaaOWaaabuaeaadaWcaaqaaiabigdaXaqaaiabcIcaOiabigdaXiabgUcaRiabdkfasjabdkfasnaaCaaaleqabaGaeGOmaidaaOGaeiykaKcaamaadmaabaWaaSaaaeaaiiGacqWFciITaeaacqWFciITcqWGHbqydaWgaaWcbaGaeGimaadabeaaaaGccuWG5bqEgaqcaiabcIcaOiabdIha4naaBaaaleaacqWGPbqAaeqaaOGaeiilaWIaemyyae2aaSbaaSqaaiabicdaWaqabaGccqGGSaalcqWGHbqydaWgaaWcbaGaeGymaedabeaakiabcMcaPaGaay5waiaaw2faaaWcbaGaemyAaKgabeqdcqGHris5aOWaamWaaeaadaWcaaqaaiab=jGi2cqaaiab=jGi2kabdggaHnaaBaaaleaacqaIXaqmaeqaaaaakiqbdMha5zaajaGaeiikaGIaemiEaG3aaSbaaSqaaiabdMgaPbqabaGccqGGSaalcqWGHbqydaWgaaWcbaGaeGimaadabeaakiabcYcaSiabdggaHnaaBaaaleaacqaIXaqmaeqaaOGaeiykaKcacaGLBbGaayzxaaGaaCzcaiaaxMaacqaIXaqmcqaI2aGnaaa@866F@

Ignoring constant factors, both the gradient vector and the Hessian matrix for robust nonlinear regression differs from the corresponding values in least-squares regression by the factor 1/(1+RR^2^). This ensures that data points far from the curve, with large values of RR, have little influence on the curve fit.

Changing the definitions of the vector of partial derivatives, Hessian matrix, and the merit score, was not enough to make our robust method work consistently. We had to make one other modification to the Marquardt-Levenberg method. After every iteration, the new goodness-of-fit merit score is compared to the one from the prior iteration. If the new one is better, then the algorithm goes on to the next iteration. If the fit is worse, then the new parameter values are rejected, and the algorithm goes back to the prior set of parameter values and decreases the step size before trying again. But with robust fitting, moving from one iteration to the next changes the value of RSDR (the new parameter values create a new curve, and so a new set of residuals). Since the value of RSDR is used in calculating the goodness-of-fit merit score, the two merit functions are not quite comparable, so their difference can not be used to reliably determine whether or not the most recent iteration improved the fit.

This problem is easy to solve. After each iteration, recompute the goodness-of-fit of the prior iteration using the new value of RSDR. Now the two goodness-of-fit scores are comparable, and can be used to decide whether the most recent iteration improved or worsened the fit.

This extra step is essential. Without it, we found that the method will not reliably converge on a reasonable best-fit curve. Unfortunately, it appears to be impossible to program this step into general purpose statistics packages that let you enter your own loss function. Therefore, implementation of the robust fitting algorithm described here requires custom programming.

### Detecting outliers

#### A brief explanation of the FDR method

The FDR method of Benjamini and Hochberg [5,6] is used to decide where to draw the line between 'significant' and 'not significant' results when you are testing many hypotheses at once. We adapted this method to detect outliers.

**Figure 15:**
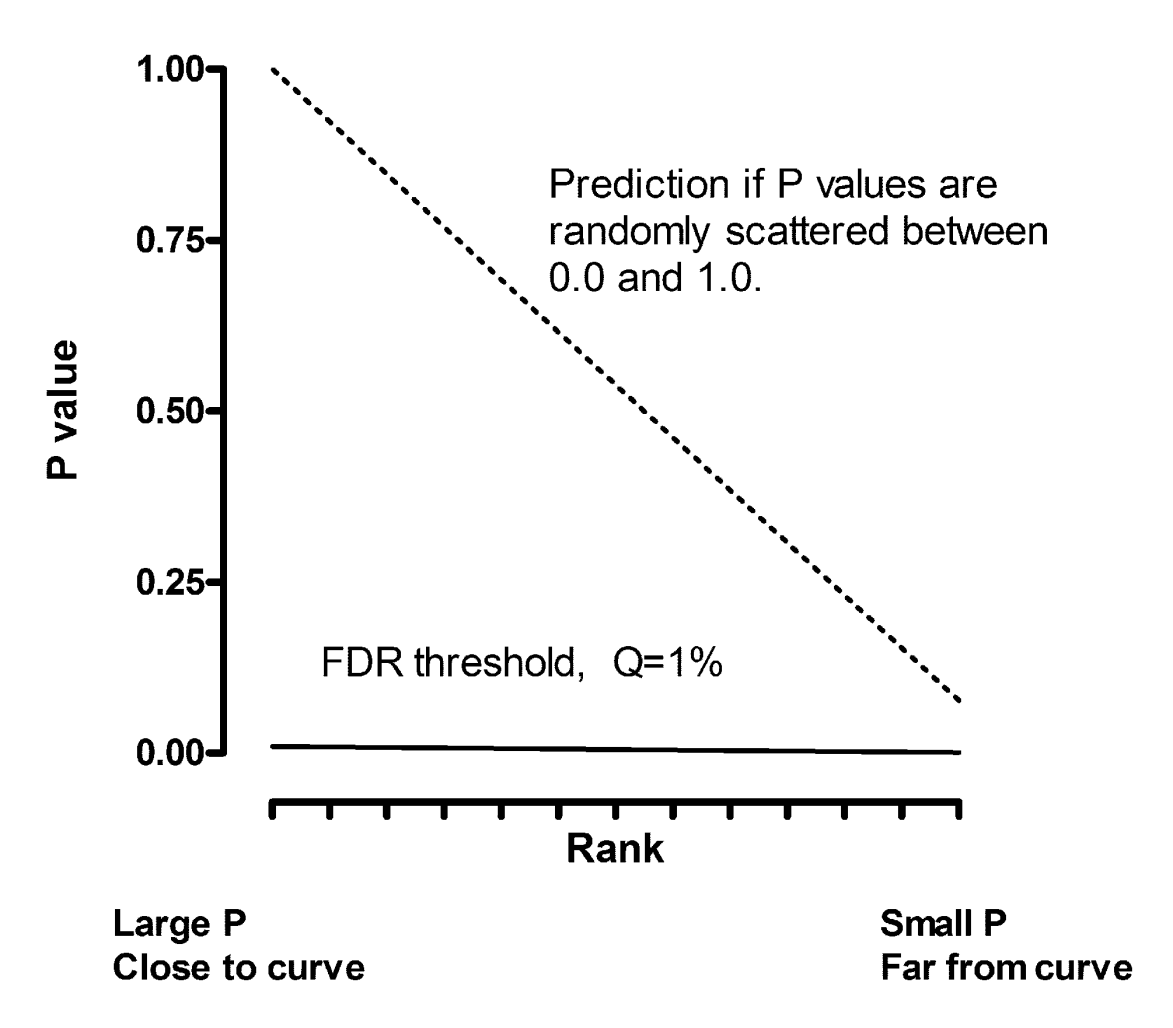
**How the Benjamini and Hochberg method works**. This method is used to decide which P values in a set of many are low enough to be defined to be 'significant'. The P values are ranked from large to small. The ranks are plotted on the X axis, with the actual P values plotted on the Y axis. The dotted line shows the expectation if in fact all null hypotheses are true – 50% of the P values are less than 0.5, 25% are less than 0.25, etc. The solid line shows the Benjamini-Hochberg threshold for declaring a P value to be significant. It is defined by multiplying the dotted line by a fraction Q (here set to 1%). When the P value is lower than that threshold, that P value and all lower P values are defined to denote 'statistically significant' differences.

The FDR method first ranks the P values from high to low, so P(1) is the largest P value and P(N) is the smallest P value. Plot these ranks (X axis) against the individual P values (Y axis). The dotted line in Figure 15 shows what you would expect to see if the P values are randomly spaced between 0 and 1. In this case, half the P values would be less than .50, one tenth would be lower than 0.10, etc. Thus the prediction line (dotted) is straight, with the largest predicted P value equalling 1.0, and the smallest predicted P value equalling 1/N.

The solid line in Figure 15 shows the threshold used to define P values as being low enough to call 'significant'. This line is created by multiplying the prediction of the dotted line by a fraction Q (whose value you pick).

The Hochberg and Benjamini method works in a stepwise fashion. It starts with the highest P value in the list and works step by step down to the smallest. At any step, if the P value is lower than the threshold, then that P value – and all lower P values – are defined to be "statistically significant".

### Applying the FDR method to detecting outliers

To use the FDR technique to detect outliers, we compute a P value for each residual testing the null hypothesis that that residual comes from a Gaussian distribution. Additionally, we restrict the maximum number of outliers that we will detect to equal 30% of N, so only compute P values for the 30% of the residuals that are furthest from the curve.

Follow these steps:

1. Fit the model using robust regression. Compute the robust standard deviation of the residuals (RSDR, defined in Equation 1).

2. Decide on a value for Q. We recommend setting Q to 1%.

3. Rank the absolute value of the residuals from low to high, so Residual_N _corresponds to the point furthest from the curve.

4. Loop from i = int(0.70*N) to N (we only test the 30% of the points furthest from the curve).

a. Compute

αi=Q(N−(i−1))N     17
 MathType@MTEF@5@5@+=feaafiart1ev1aaatCvAUfKttLearuWrP9MDH5MBPbIqV92AaeXatLxBI9gBaebbnrfifHhDYfgasaacH8akY=wiFfYdH8Gipec8Eeeu0xXdbba9frFj0=OqFfea0dXdd9vqai=hGuQ8kuc9pgc9s8qqaq=dirpe0xb9q8qiLsFr0=vr0=vr0dc8meaabaqaciaacaGaaeqabaqabeGadaaakeaaiiGacqWFXoqydaWgaaWcbaGaemyAaKgabeaakiabg2da9maalaaabaGaemyuae1aaeWaaeaacqWGobGtcqGHsislcqGGOaakcqWGPbqAcqGHsislcqaIXaqmcqGGPaqkaiaawIcacaGLPaaaaeaacqWGobGtaaGaaCzcaiaaxMaacqaIXaqmcqaI3aWnaaa@3EFE@

b. Compute

t=|Residuali|RSDR     18
 MathType@MTEF@5@5@+=feaafiart1ev1aaatCvAUfKttLearuWrP9MDH5MBPbIqV92AaeXatLxBI9gBaebbnrfifHhDYfgasaacH8akY=wiFfYdH8Gipec8Eeeu0xXdbba9frFj0=OqFfea0dXdd9vqai=hGuQ8kuc9pgc9s8qqaq=dirpe0xb9q8qiLsFr0=vr0=vr0dc8meaabaqaciaacaGaaeqabaqabeGadaaakeaacqWG0baDcqGH9aqpdaWcaaqaamaaemaabaacbiGae8NuaiLae8xzauMaem4CamNaemyAaKMaemizaqMaemyDauNaemyyaeMaemiBaW2aaSbaaSqaaiabdMgaPbqabaaakiaawEa7caGLiWoaaeaacqWGsbGucqWGtbWucqWGebarcqWGsbGuaaGaaCzcaiaaxMaacqaIXaqmcqaI4aaoaaa@466F@

c. Compute the two-tailed P value from the t distribution with N-K degrees of freedom (N is number of data points; K is number of parameters fit by nonlinear regression).

d. Test whether this P value (calculated in 4c) is less than α_i_. (calculated in 4a).

• If yes, define the data point that corresponds to Residual_i_, and all data points further from the curve, to be outliers (and stop the outlier hunt).

• If no, and if i = N, then conclude there are no outliers.

• If no, and i<N, then set i = i + 1 and loop back.

5. Delete the outliers, and run least-squares regression on the remaining points.

### Unequal weighting

So far, we have assumed that all points are weighted equally. In fact, it often makes sense to perform nonlinear regression with unequal weighting. The most common case is when the average scatter of the data around the curve is proportional to the height of the curve. The average size (absolute value) of the residuals is not consistent, but the average size of the relative residual (the absolute value of the residual divided by the Y value of the curve) is consistent. One example is when most of the experimental error is due to pipettes delivering variable volumes. In this case, the average absolute value of the residual increases as Y increases, but remains a consistent percentage of the height of the curve. In this case, ordinary nonlinear regression would minimize this weighted sum-of-squares:

Weighted Gaussian Merit=∑i[yi−y^(xi,a0,a1)y^(xi,a0,a1)]2     19
 MathType@MTEF@5@5@+=feaafiart1ev1aaatCvAUfKttLearuWrP9MDH5MBPbIqV92AaeXatLxBI9gBaebbnrfifHhDYfgasaacH8akY=wiFfYdH8Gipec8Eeeu0xXdbba9frFj0=OqFfea0dXdd9vqai=hGuQ8kuc9pgc9s8qqaq=dirpe0xb9q8qiLsFr0=vr0=vr0dc8meaabaqaciaacaGaaeqabaqabeGadaaakeaacqqGxbWvcqqGLbqzcqqGPbqAcqqGNbWzcqqGObaAcqqG0baDcqqGLbqzcqqGKbazcqqGGaaicqqGhbWrcqqGHbqycqqG1bqDcqqGZbWCcqqGZbWCcqqGPbqAcqqGHbqycqqGUbGBcqqGGaaicqqGnbqtcqqGLbqzcqqGYbGCcqqGPbqAcqqG0baDcqGH9aqpdaaeqbqaamaadmaabaWaaSaaaeaacqWG5bqEdaWgaaWcbaGaemyAaKgabeaakiabgkHiTiqbdMha5zaajaGaeiikaGIaemiEaG3aaSbaaSqaaiabdMgaPbqabaGccqGGSaalcqWGHbqydaWgaaWcbaGaeGimaadabeaakiabcYcaSiabdggaHnaaBaaaleaacqaIXaqmaeqaaOGaeiykaKcabaGafmyEaKNbaKaacqGGOaakcqWG4baEdaWgaaWcbaGaemyAaKgabeaakiabcYcaSiabdggaHnaaBaaaleaacqaIWaamaeqaaOGaeiilaWIaemyyae2aaSbaaSqaaiabigdaXaqabaGccqGGPaqkaaaacaGLBbGaayzxaaaaleaacqWGPbqAaeqaniabggHiLdGcdaahaaWcbeqaaiabikdaYaaakiaaxMaacaWLjaGaeGymaeJaeGyoaKdaaa@72DB@

More generally, define w_i _to be a weighting factor:

Weighted Gaussian Merit=∑i(yi−y^(xi,a0,a1))2Wi     20
 MathType@MTEF@5@5@+=feaafiart1ev1aaatCvAUfKttLearuWrP9MDH5MBPbIqV92AaeXatLxBI9gBaebbnrfifHhDYfgasaacH8akY=wiFfYdH8Gipec8Eeeu0xXdbba9frFj0=OqFfea0dXdd9vqai=hGuQ8kuc9pgc9s8qqaq=dirpe0xb9q8qiLsFr0=vr0=vr0dc8meaabaqaciaacaGaaeqabaqabeGadaaakeaacqqGxbWvcqqGLbqzcqqGPbqAcqqGNbWzcqqGObaAcqqG0baDcqqGLbqzcqqGKbazcqqGGaaicqqGhbWrcqqGHbqycqqG1bqDcqqGZbWCcqqGZbWCcqqGPbqAcqqGHbqycqqGUbGBcqqGGaaicqqGnbqtcqqGLbqzcqqGYbGCcqqGPbqAcqqG0baDcqGH9aqpdaaeqbqaamaalaaabaWaaeWaaeaacqWG5bqEdaWgaaWcbaGaemyAaKgabeaakiabgkHiTiqbdMha5zaajaGaeiikaGIaemiEaG3aaSbaaSqaaiabdMgaPbqabaGccqGGSaalcqWGHbqydaWgaaWcbaGaeGimaadabeaakiabcYcaSiabdggaHnaaBaaaleaacqaIXaqmaeqaaOGaeiykaKcacaGLOaGaayzkaaWaaWbaaSqabeaacqaIYaGmaaaakeaacqWGxbWvdaWgaaWcbaGaemyAaKgabeaaaaaabaGaemyAaKgabeqdcqGHris5aOGaaCzcaiaaxMaacqaIYaGmcqaIWaamaaa@682E@

When scatter is not uniform, we found that it does not make sense to include weighting factors in robust regression, either by weighting the residuals or by weighting the robust merit function itself. In either case, simulations showed that the use of weighting factors reduced the accuracy of the fits, because outliers with very low Y values got extra weight, and so influenced the curve too much. Therefore we suggest always performing robust regression without considering weighting factors.

Weighting factors should be considered when identifying outliers. This is done by computing the weighted residuals using Equation 22:

Weighted Residuali=|yi−y^(xi,a0,a1)|y^(xi,a0,a1)=DWi     22
 MathType@MTEF@5@5@+=feaafiart1ev1aaatCvAUfKttLearuWrP9MDH5MBPbIqV92AaeXatLxBI9gBaebbnrfifHhDYfgasaacH8akY=wiFfYdH8Gipec8Eeeu0xXdbba9frFj0=OqFfea0dXdd9vqai=hGuQ8kuc9pgc9s8qqaq=dirpe0xb9q8qiLsFr0=vr0=vr0dc8meaabaqaciaacaGaaeqabaqabeGadaaakeaacqqGxbWvcqqGLbqzcqqGPbqAcqqGNbWzcqqGObaAcqqG0baDcqqGLbqzcqqGKbazcqqGGaaicqqGsbGucqqGLbqzcqqGZbWCcqqGPbqAcqqGKbazcqqG1bqDcqqGHbqycqqGSbaBdaWgaaWcbaacbiGae8xAaKgabeaakiabg2da9maalaaabaWaaqWaaeaacqWG5bqEdaWgaaWcbaGaemyAaKgabeaakiabgkHiTiqbdMha5zaajaGaeiikaGIaemiEaG3aaSbaaSqaaiabdMgaPbqabaGccqGGSaalcqWGHbqydaWgaaWcbaGaeGimaadabeaakiabcYcaSiabdggaHnaaBaaaleaacqaIXaqmaeqaaOGaeiykaKcacaGLhWUaayjcSdaabaGafmyEaKNbaKaacqGGOaakcqWG4baEdaWgaaWcbaGaemyAaKgabeaakiabcYcaSiabdggaHnaaBaaaleaacqaIWaamaeqaaOGaeiilaWIaemyyae2aaSbaaSqaaiabigdaXaqabaGccqGGPaqkaaGaeyypa0ZaaSaaaeaacqWGebaraeaadaGcaaqaaiabdEfaxnaaBaaaleaacqWGPbqAaeqaaaqabaaaaOGaaCzcaiaaxMaacqaIYaGmcqaIYaGmaaa@6E75@

From the list of weighted residuals, calculate the P68 and the weighted RSDR from Equation 1. Outlier identification then works exactly as already explained. After any outliers are removed, the data are fit using weighed least-squares regression that minimizes Equation 20.

### Worked example. Data with an outlier

In this section, we'll show in detail how the example shown at the beginning of the paper is analyzed. The data show a signal measured at various time points, fit to a single-phase exponential decay. We are fitting three parameters: the Y value at time 0, the rate constant, and the plateau.

**Figure 16:**
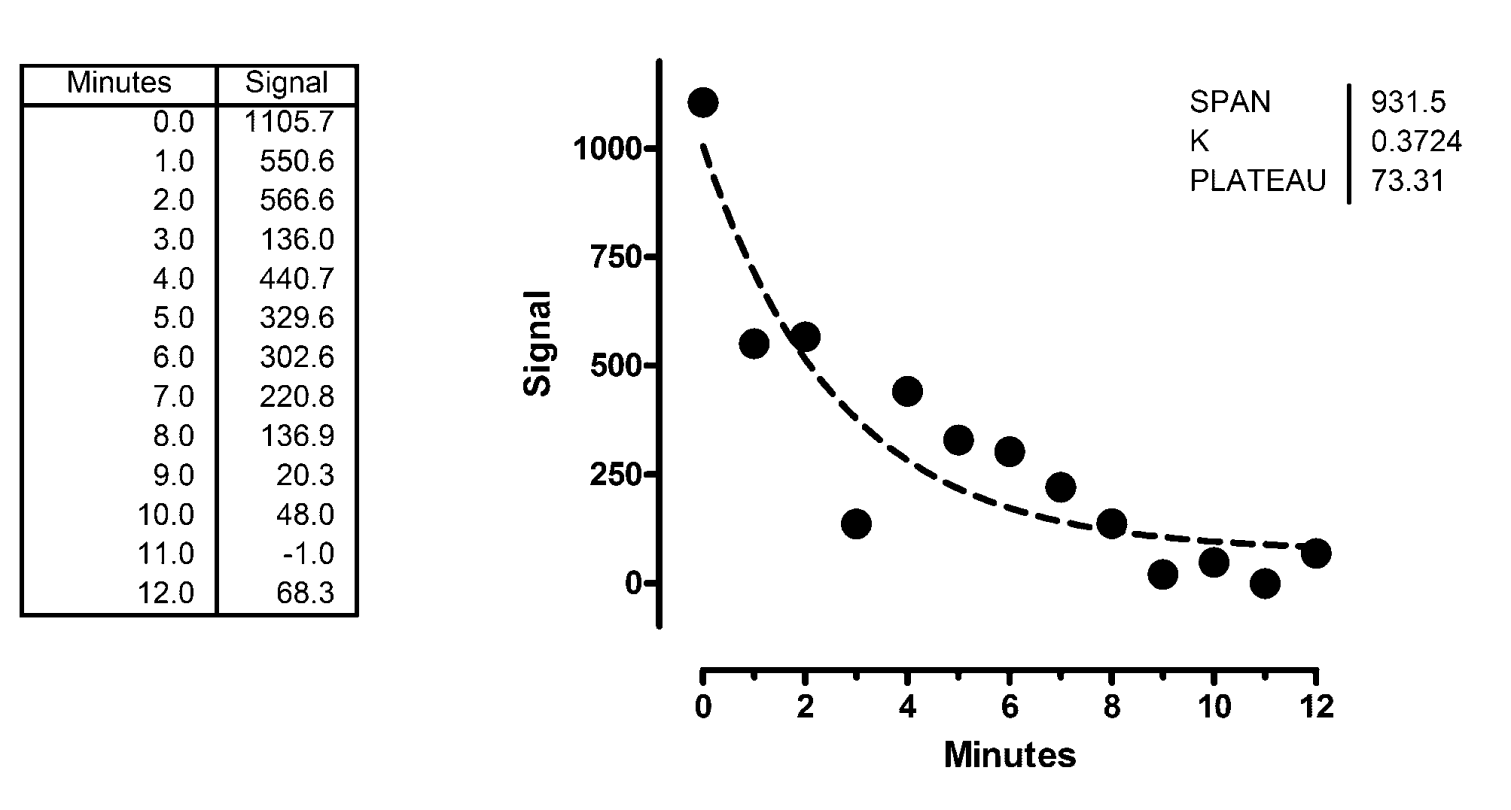
**Worked example. Data and least-squares fit**. The dashed line shows the results of least-squares regression to a one-phase exponential decay model.

We start with the least-squares fit (Fig. 16).

**Figure 17:**
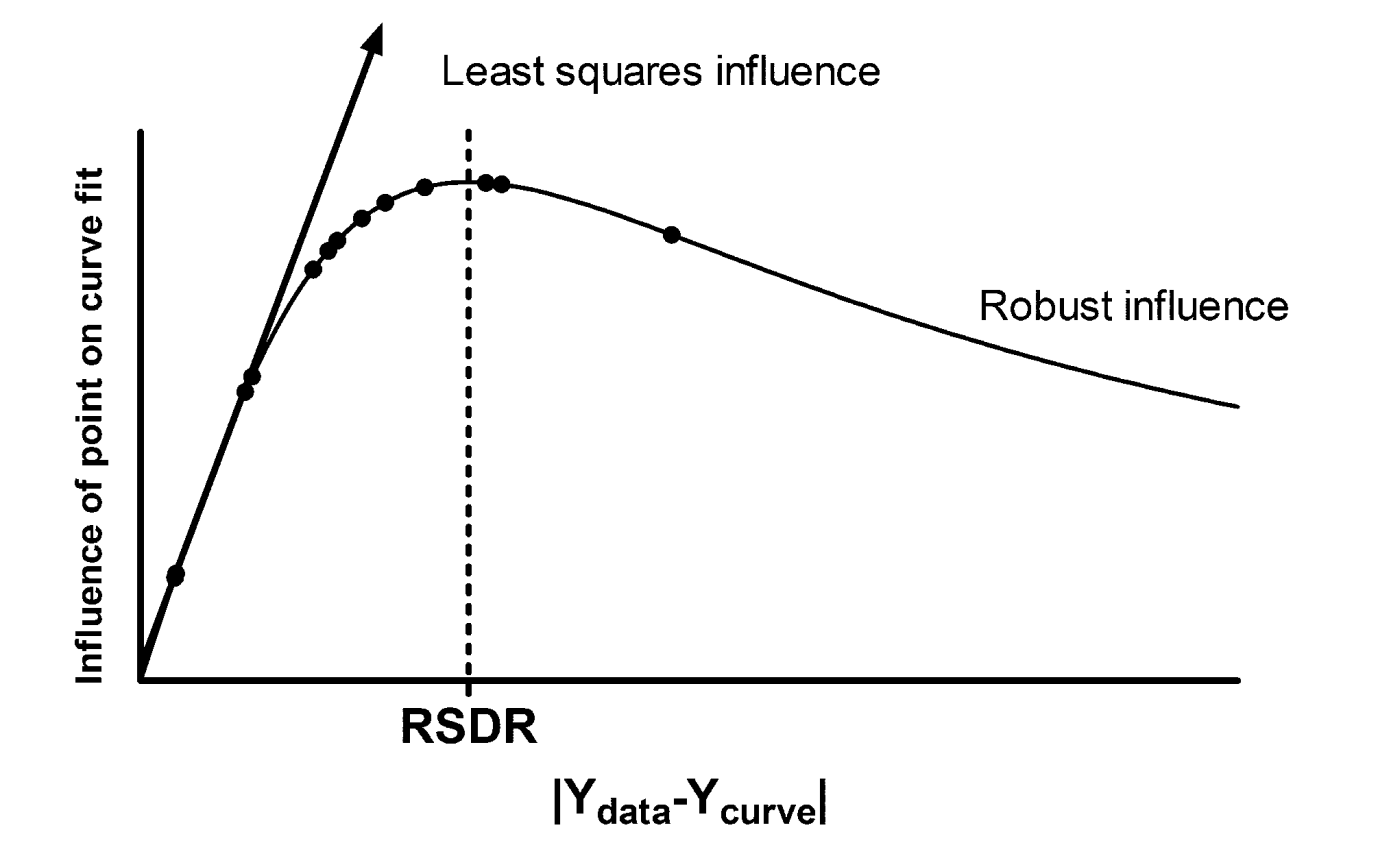
**Worked example. The influence function for robust fitting, prior to the first iteration**. The influence function is defined as RR/(1+RR^2^), where RR is defined in Equation 13. Even the points with the largest residuals (to the right on the graph) have substantial influence.

The RSDR is 153.5. Figure 17 shows influence function at the beginning of robust regression. Even the point with the largest residual has quite a bit of influence.

**Figure 18:**
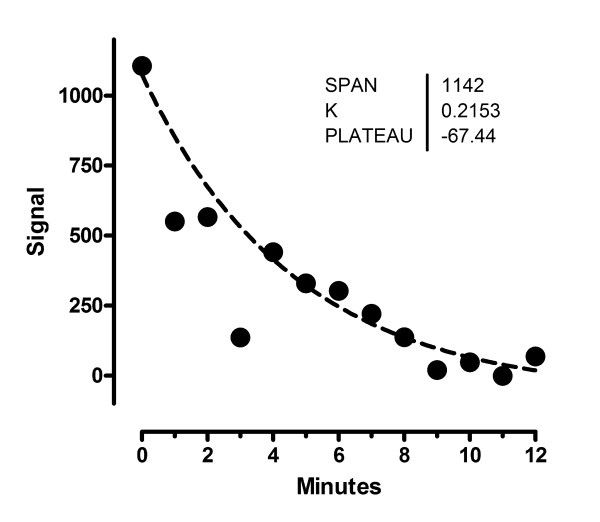
Worked example. Fit with robust nonlinear regression.

Figure 18 shows the result at the end of robust fitting. Now the outliers are relatively ignored, so the curve comes closer to the bulk of the points. The RSDR has come down to 78.24.

**Figure 19:**
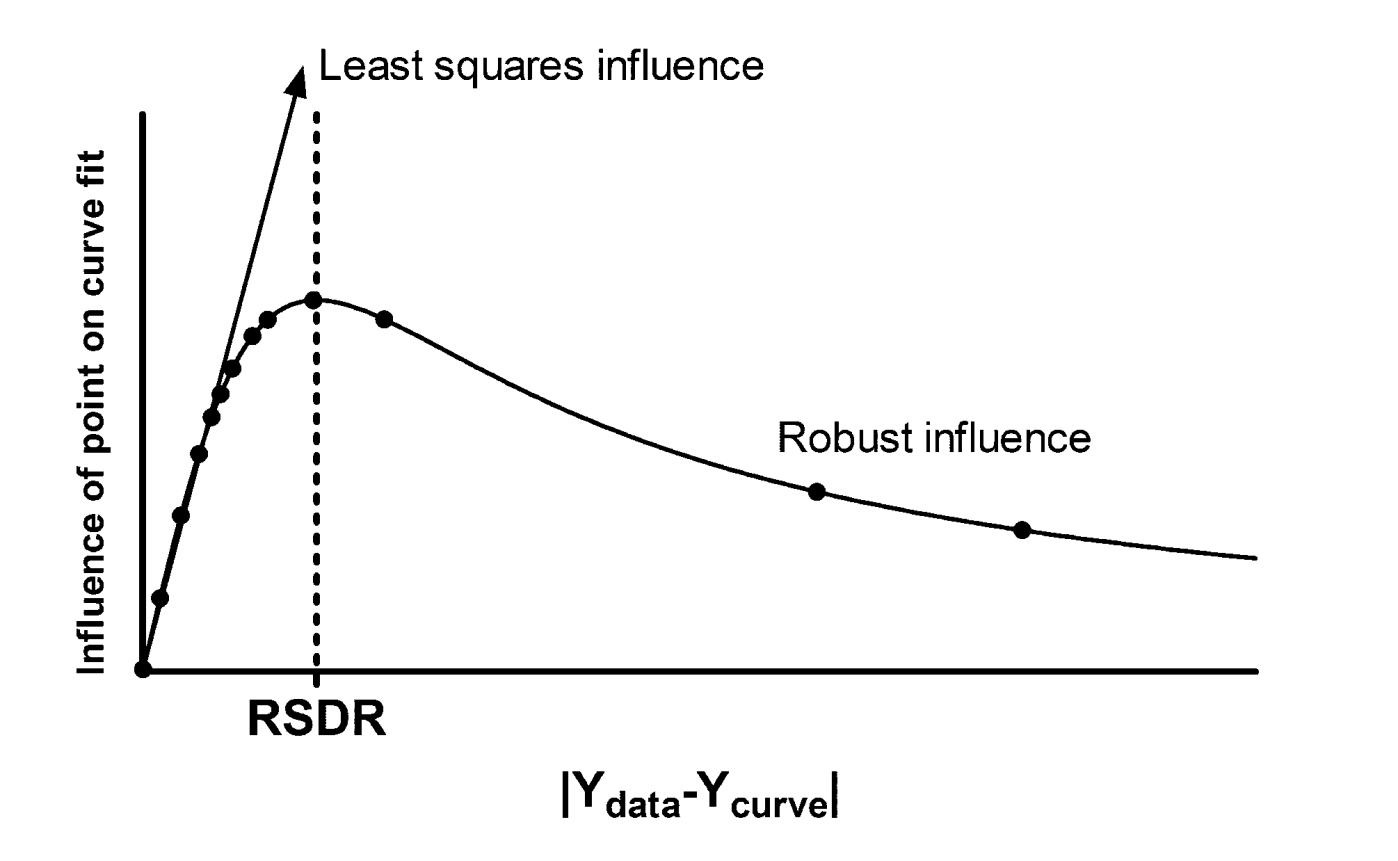
**Worked example. The influence function for robust fitting, after the final iteration**. Now that the curve is much closer to most of the points, the RSDR is lower, so the influence curve is shifted to the left. This makes two of the points (to the right) have much less influence than they had at the beginning (compare to Figure 17).

Figure 19 shows influence curve at the end of robust fitting. Note that two of the points have much less influence than they had at the beginning of robust regression. As the robust regression proceeds, the curve gets closer to the bulk of the points, the RSDR goes down, so the outlying points get less influence.

Now let's determine which points are outliers. For each point, we compute the t ratio as the absolute value of the residual divided by the RSDR, and then use that to determine a P value from the t distribution with 10 degrees of freedom. We then sort the values by P value, and compute the threshold P value for each point as Q* [N- (i-1)]/N. Table 1 shows these thresholds computed for two different values of Q.

If you set Q to 5%, the second to last row in the table is the first row where the P value is less than the threshold. Therefore, define that point to be an outlier, as well as all points with smaller P values (in this case, just the point defined by the last row).

If you set Q to 1% (our recommendation), only the point at time = 3 has a P value less than the threshold, so only that one point is eliminated as an outlier.

**Figure 20:**
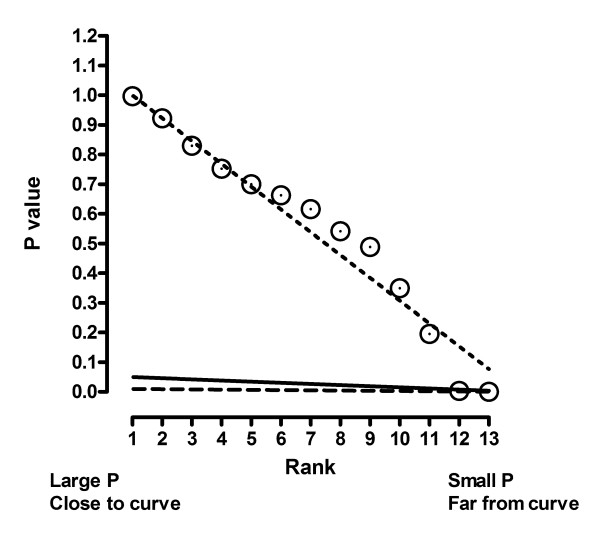
**Worked example. Using the Benjamini and Hochberg method to detect outliers**. A P value was determined for each point by computing a t ratio by dividing its residual by the RSDR, and computing a two-tailed P value from the t distribution. See Table 1. The P values are shown plotted against their rank. The dashed line shows what you'd expect to see if the P values are randomly scattered between 0 and 1. All but lowest two of the P values lie very close to this line. The solid line shows the cutoff when Q is set to 5%. Both of the points with the lowest P values (the two points furthest from the robust best-fit curves) are defined to be outliers. The dashed line shows the cutoff when Q is set to 1% as we suggest. Only one point is an outlier with this definition, which we choose to use.

Outlier elimination is best understood graphically (Fig. 20). The X axis plots the rank of the P values, with rank 1 being the largest P value (corresponding to the point closest to the curve) on the left. The Y axis plots the actual P values. Each P value tests whether a point is an outlier by testing the null hypothesis that the point came from a Gaussian distribution with a mean of zero, a standard deviation equal to the RSDR, and N-K (13 - 3 = 10) degrees of freedom. The dashed line shows what you'd expect to see if all scatter is Gaussian.

The solid line shows the cut-off defined by the False Discovery Rate setting Q equal to 5%. Scanning left to right, find the first point that is below this line. That point, and all points further to the right are defined to be outliers. In this example, the last two points on the right of the graph are below the Q = 5% line. The dashed line shows the cutoff defined by Q equal to 1%. Only the last point is below it.

**Figure 21:**
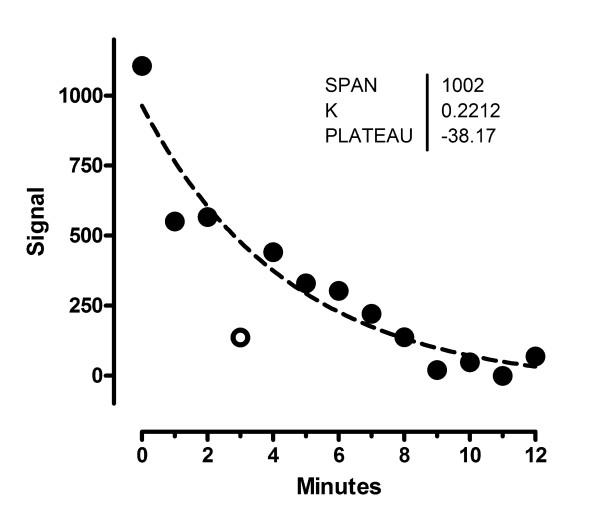
**Worked example. Least squares regression after excluding the outlier**. The outlier is shown with an open symbol. It was not included in the least squares regression (dashed curve).

Figure 21 shows the least-squares fit of the remaining data points after eliminating the outlier (defining outliers with Q = 1%, there is only one). The outlier is shown as an open circle but is not included in the regression calculations.

### Worked example. Data with no outliers

**Figure 22:**
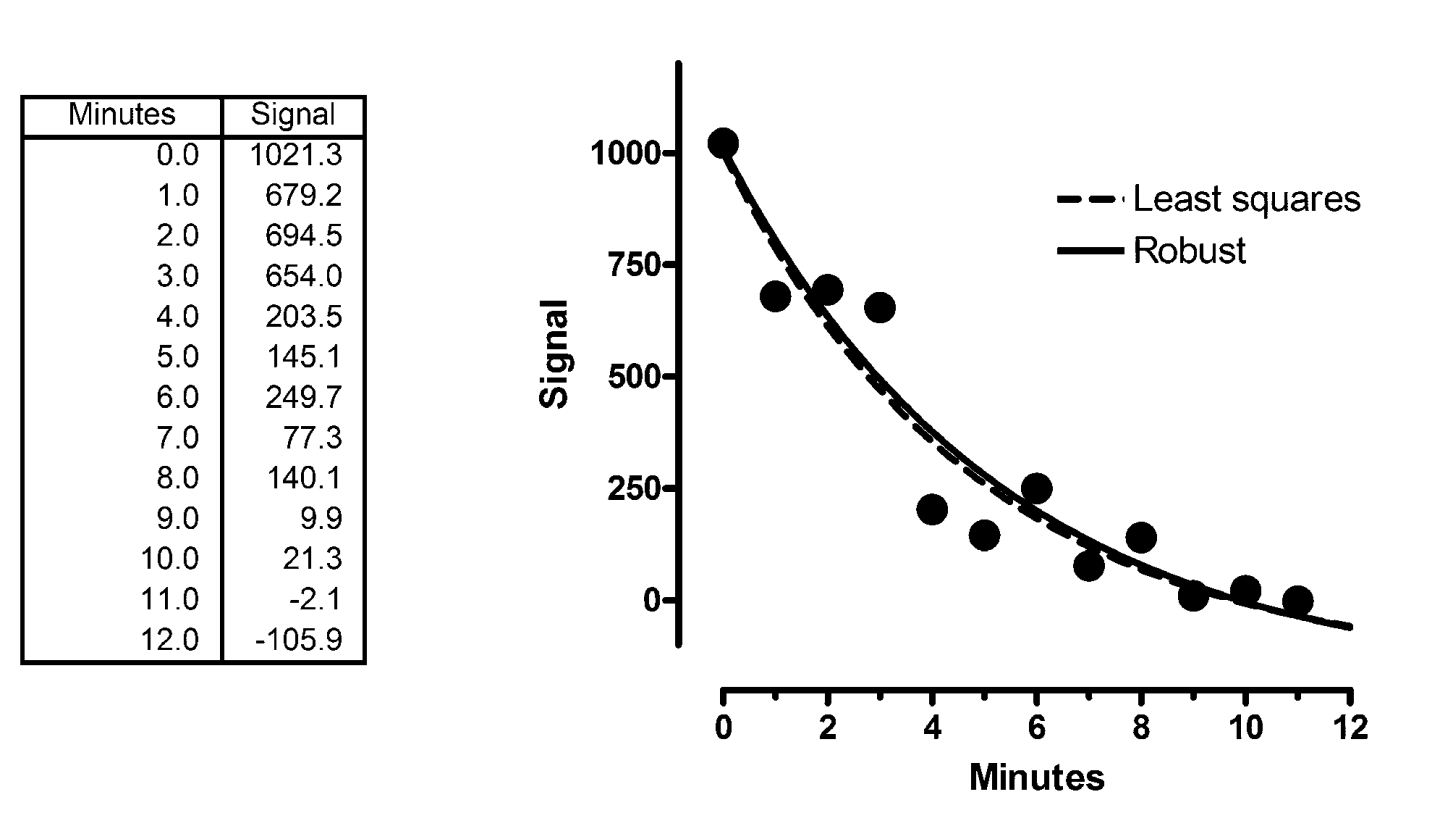
**A second example**. The least squares fit (dashed) and robust fit (solid) are almost identical.

Figure 22 shows a second example. The least squares fit is in dashed curve, and the robust fit in solid (RSDR = 95.6). The two are almost indistinguishable.

**Figure 23:**
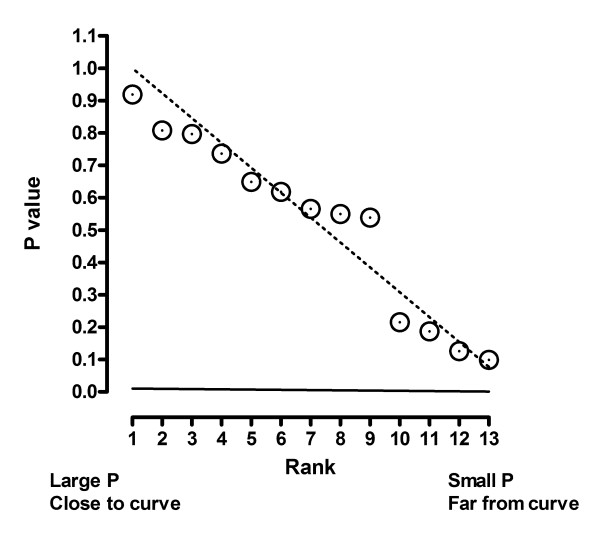
**Using the Benjamini and Hochberg method to detect outliers in the second example**. A P value was determined for each point by computing a t ratio by dividing its residual by the RSDR, and computing a two-tailed P value from the t distribution. The P values are shown plotted against their rank. The dashed line shows what you'd expect to see if the P values are randomly scattered between 0 and 1. All the points are near this line, and none are below the solid Q = 1% threshold line. Therefore none of the points are defined to be outliers.

Figure 23 shows the distribution of P values. The actual distribution of P values is very close to the predicted distribution. The solid line shows the cutoff defined by Q = 1%. None of the P values are less than this threshold, so none of the points are declared to be significant outliers. Even if we increased Q to 5%, none of the points would be designated as outliers. We used robust regression to define a baseline from which to look for outliers. We didn't find any, so report the results of least-squares regression of all the data. For this example, therefore, the results of our method are identical in every way to standard least-squares nonlinear regression.

## Authors' contributions

The two authors contributed nearly equally to the work. HM developed the outlier elimination method, did the simulations, and wrote the first draft of the manuscript. RB developed the robust fitting and helped polish the manuscript. Both authors read and approved the final manuscript.

## Figures and Tables

**Table 1:** Worked example. Which points are outliers?

**Time(X)**	**Residual**	**t ratio**	**P value**	**Threshold Q = 5%**	**Threshold Q = 1%**
8	0.31	0.00	0.9969	0.0500	0.0100
5	7.85	0.10	0.9221	0.0462	0.0092
10	-17.26	0.22	0.8298	0.0423	0.0085
4	25.38	0.32	0.7524	0.0385	0.0077
0	31.05	0.40	0.6999	0.0346	0.0069
7	35.16	0.45	0.6628	0.0308	0.0062
11	-40.49	0.52	0.6160	0.0269	0.0054
12	49.48	0.63	0.5413	0.0231	0.0046
6	56.23	0.72	0.4888	0.0193	0.0038
9	-76.82	0.98	0.3494	0.0154	0.0031
2	-108.51	1.39	0.1956	0.0116	0.0023
1	-302.88	3.87	0.0031	0.0077	0.0015
3	-395.21	5.05	0.0005	0.0039	0.0008
